# Bioconversion of Fish Discards through the Production of Lactic Acid Bacteria and Metabolites: Sustainable Application of Fish Peptones in Nutritive Fermentation Media

**DOI:** 10.3390/foods9091239

**Published:** 2020-09-04

**Authors:** José Antonio Vázquez, Ana I. Durán, Araceli Menduíña, Margarita Nogueira, Ana María Gomes, Joana Antunes, Ana Cristina Freitas, Esther Dagá, Paula Dagá, Jesus Valcarcel

**Affiliations:** 1 Grupo de Biotecnología y Bioprocesos Marinos, Instituto de Investigaciones Marinas (IIM-CSIC), C/Eduardo Cabello, 6, CP 36208 Vigo, Galicia, Spain; anais@iim.csic.es (A.I.D.); araceli@iim.csic.es (A.M.); marga@iim.csic.es (M.N.); jvalcarcel@iim.csic.es (J.V.); 2 Laboratorio de Reciclado y Valorización de Materiales Residuales (REVAL), Instituto de Investigaciones Marinas (IIM-CSIC), C/Eduardo Cabello, 6, CP 36208 Vigo, Galicia, Spain; 3 CBQF-Centro de Biotecnologia e Química Fina—Laboratório Associado, Escola Superior de Biotecnologia, Universidade Católica Portuguesa, Rua Diogo Botelho 1327, 4169-005 Porto, Portugal; amgomes@porto.ucp.pt (A.M.G.); jch.antunes@gmail.com (J.A.); afreitas@porto.ucp.pt (A.C.F.); 4 Bialactis Biotech S.L., Grupo Zendal, Lugar a Relva, S/N, CP 36410 O Porriño, Pontevedra, Galicia, Spain; esther@bialactis.com (E.D.); paula@bialactis.com (P.D.)

**Keywords:** fish discards valorization, lactic acid bacteria production, marine peptones, microbial bioconversion, sustainability, logistic equation

## Abstract

In the current work, we study the capacity of 30 peptones obtained by enzyme proteolysis of ten discarded fish species (hake, megrim, red scorpionfish, pouting, mackerel, gurnard, blue whiting, Atlantic horse mackerel, grenadier, and boarfish) to support the growth and metabolite production of four lactic acid bacteria (LAB) of probiotic and technological importance. Batch fermentations of *Lactobacillus plantarum*, *L. brevis*, *L. casei*, and *Leuconostoc mesenteroides* in most of the media formulated with fish peptones (87% of the cases) led to similar growths (quantified as dry-weight biomass and viable cells) and metabolites (mainly lactic acid) than in commercial control broth (MRS). Comparisons among cultures were performed by means of the parameters obtained from the mathematical fittings of experimental kinetics to the logistic equation. Modelling among experimental and predicted data from each bioproduction was generally accurate. A simple economic assessment demonstrated the profitability achieved when MRS is substituted by media formulated with fish discards: a 3–4-fold reduction of costs for LAB biomass, viable cells formation, and lactic and acetic acid production. Thus, these fish peptones are promising alternatives to the expensive commercial peptones as well as a possible solution to valorize discarded fish biomasses and by-products.

## 1. Introduction

The current European Union fishery policy, aimed at gradually eliminating fish discards, requires fishing vessels to land all catches of regulated commercial species. The unwanted catches landed cannot be directly sold for human consumption, being considered a byproduct [1]. As a result, the Landing Obligation (LO) policy will lead to an increasing amount of new biomass of discarded fish in European ports. While a fraction of this biomass may be suitable for the production of processed fish products, undersized and low quality individuals require alternative plans of valorization in order to: (1) avoid the pollution problems associated with its presence in fishing ports (health risks by microbial fish spoilage); (2) reduce the economical negative effect that the LO will generate to the fishing sector; and (3) obtain bioproducts of high-added value beyond the conventional fish meal production.

A number of valorization strategies exist to recover valuable materials from this last fraction, as well as from processing by-products [2,3,4]. Whenever food applications are not possible, the obtention of bioproducts is usually the preferred option. Depending on the characteristics of the raw material, technical parameters, market, and economic aspects, a number of bioproducts may represent viable valorization options [2]. These include polysaccharides (chondroitin sulfate, chitin, and chitosan) [5,6], lipids (polyunsaturated fatty acids, carotenoids) [7,8], minerals [9], and protein (bioactive peptides, enzymes, collagen and gelatin and peptones) [10,11,12,13,14]. Only certain species and particular tissues contain, or are sufficiently rich, in some of these bioproducts, whereas proteinaceous material is ubiquitous in fish.

In this regard, valorization processes to hydrolyze protein represent a viable way to recover bioactive peptides [11], essential amino acids valuable in the formulation of aquaculture feed [15], and amino acids and soluble peptides as an organic nitrogen source in culture media [14]. The latter, commercially known as peptones, are the most expensive nitrogen source in microbial culture, considerably impacting production costs in the biotechnological industries [16,17]. Therefore, the development of cheaper peptone sources from waste, such as fish discards, may influence the economic viability of such bioprocesses.

Different methods have been shown as capable of producing peptones from fish by-products, whole individuals, and industrial effluents. These protocols include enzymatic hydrolysis with commercial or endogenous proteases, acid and alkaline hydrolysis, and also boiling [14,18,19,20,21]. The performance of the peptones produced are in most cases evaluated by the culture of lactic acid bacteria, as these are extremely fastidious microorganisms with complex organic substrate requirements [22]. Furthermore, fermentation of lactic acid bacteria yields relevant products, such as prebiotics, probiotics, bacteriocins, polysaccharides, and of course, lactic acid [23,24,25,26].

The present study aims at providing a comprehensive outlook on the feasibility of marine peptones obtained from a wide range of fish discards as nitrogen sources for the growth of lactic acid bacteria (LAB) with industrial relevance. To this end, we produced peptones from ten discarded species, including fish heads and skins, as by-products from fish mince production, as well as whole specimens. As a result of the enzymatic hydrolysis of each substrate, we produced thirty different peptones, testing their performance with four species of lactic acid bacteria commonly used in the industry for the fermentation of vegetables, milk, meat, and as probiotics.

## 2. Materials and Methods

### 2.1. Fish Discards and Production of Marine Peptones

**Figure 1:**
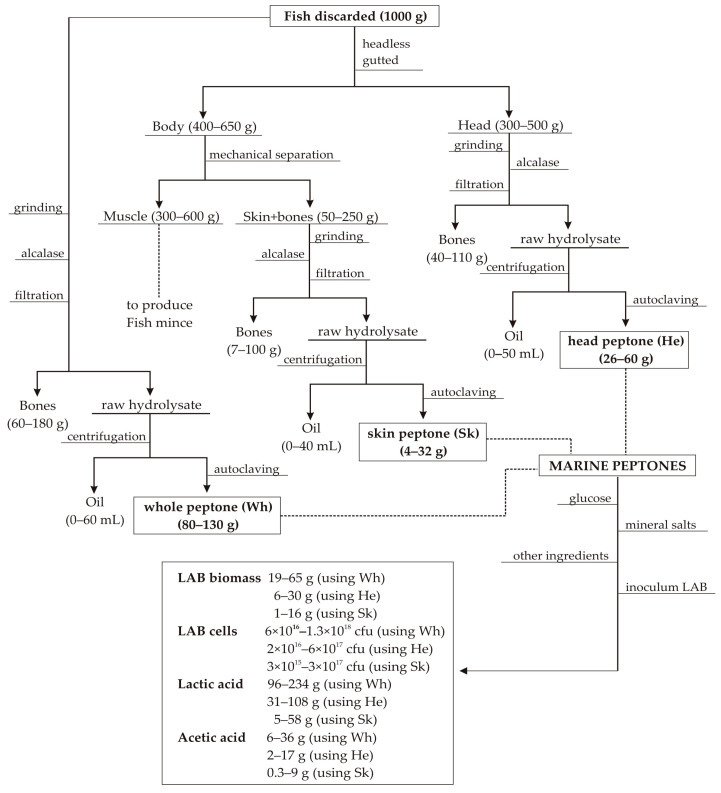
Flowchart of steps involved in the production of peptones from skin, head, and whole individuals of discarded fish by fishing fleets, and their application for the production of LAB and metabolites. The mass balances, employing 1000 g of fish discarded as calculation basis, are also included.

The fish discards used in this study were grenadier (Gr, *Macrourus* sp.), megrim (Me, *Lepidorhombus boscii*), European hake (Ha, *Merluccius merluccius*), boarfish (Bo, *Capros aper*), Atlantic horse mackerel (AHM, *Trachurus trachurus*) blue whiting (BW, *Micromesistius poutassou*), mackerel (Ma, *Scomber scombrus*), red scorpionfish (RS, *Scorpaena scrofa*), pouting (Po, *Trisoreptus luscus*), and gurnard (Gu, *Trigla* spp.). These species were the most abundant discarded fish by Galician fishing fleets [27], they were selected on board, in different fishing days, and maintained at −20 °C. After landing, one part of the discards (around 4–5 kg of each species) was kept at −18 °C until processing and the remaining (around 20–30 kg of each species) was manually decapitated and gutted. This last fraction was mechanically processed, in a bone separator (Baader 694, Germany), to recover fish mince [28] and generating a mixture of skins and bones. The substrates studied as the source of peptones were heads (He), the mixture of skins and bones (Sk), and the whole specimens (Wh) previously frozen (Figure 1).

He, Sk, and Wh were ground in a meat grinder and stored at −18 °C. Enzymatic hydrolysis of these materials were performed in a controlled pH-Stat system with a 5 L glass-reactor (mixing 1 kg of milled discards with 2 L of distilled water, (S:L) ratio of 1:2 *w*/*v*). Alcalase 2.4 L (Novozymes, Nordisk, Bagsvaerd, Denmark) was the commercial protease applied at 1% (*v*/*w*), pH 8.65, 200 rpm of agitation and 60 °C for 4 h [4,14,27]. Bones were removed from raw hydrolysates by filtering and the liquid fraction was centrifuged (15,000× *g*/20 min) to separate oil and hydrolysates. The marine peptones were obtained after autoclaving (101 °C/60 min) these hydrolysates. In Figure 1, a scheme of this procedure is represented. The soluble proteins and the reducing and total sugar contents are shown in Table 1.

### 2.2. Bacterial Protocols and Culture Media

Four lactic acid bacteria from CECT (Spanish Type Culture Collection) were assayed: *Lactobacillus brevis* CECT 4043 (Lb 1), *Lactobacillus plantarum* CECT 220 (Lb 2), *Lactobacillus casei* CECT 475 (Lb 3), and *Leuconostoc mesenteroides* ssp. *mesenteroides* CECT 4046 (Ln). Stock cultures were preserved at −80 °C in Man, Rogosa, and Sharpe medium (MRS, from Pronadisa, Hispanlab S.A., Spain) with 25% glycerol (*w*/*w*). The concentration of soluble protein in the alternative media (with peptones from fish discards) was established by substituting the Lowry protein content in commercial MRS (10 g/L from meat extract and bactopeptone). Initial concentration of glucose (reducing sugars) was fixed at 24 g/L. Fermentations were carried out in duplicate using 300 mL Erlenmeyer flasks with 180 mL of medium at 30 °C (Lb 1, Lb 2, and Ln) and 35 °C (Lb 3) at 200 rpm on an orbital shaker. In all cases, the initial pH was adjusted to 6.0 with 5 N NaOH and culture broths were finally sterilized separately at 121 °C for 15 min. Inocula (0.5% *w*/*v*) consisted of cellular suspensions from 12−16 h cultures in control medium.

### 2.3. Analytical Determinations

Samples from each culture were collected at pre-determined times and divided into two aliquots. The first one was employed in the quantification of the viable cells using plate count in MRS agar medium. Serial tenfold dilutions were prepared in peptone-buffered solutions, and 0.1 mL samples were extended in plate by triplicate, incubated at 30 °C for 48 h, and manually counted. The obtained results were expressed as G = ln(N/N_0_), where N is the colony-forming units per mL (cfu/mL) and N_0_ is the initial colony-forming units per mL (cfu/mL). The second aliquot was centrifuged at 3273× *g* for 15 min, from which the supernatant was used for determining the content of soluble proteins, reducing sugars, and lactic and acetic acids. The sediment was washed and resuspended in distilled water at an appropriate dilution to measure the optical density (OD) at 700 nm and then the dry weight was estimated from a calibration curve (OD vs. dry weight).

Marine peptones and microbial postincubated were analyzed, in duplicate, as follows: (1) Reducing sugars (RS) by 3,5-dinitrosalicylic reaction [29]; (2) total soluble proteins (Pr) using Lowry method [30]; (3) total sugars (TS) by Dubois et al. protocol [31], and (4) organic acids (lactic and acetic) by HPLC [32].

### 2.4. Mathematical Modelling of Bacterial Kinetics

The experimental bioproductions, bacterial biomass as dry weight (X), cell formation (G), lactic acid production (L), and acetic acid production (A), were modelled by the following logistic equation [33]:

(1) $$P=\frac{P_{m}}{1+\exp\left[2+\frac{4v_{P}}{P_{m}}\left(\lambda_{P}-t\right)\right]}$$

Other parameters from Equation (1) were additionally determined in order to know the rest of the sigmoid experimental profile [33]:

(2) $$\mu_{P}=\frac{4v_{P}}{P_{m}}$$

(3) $$\tau_{P}=\lambda_{P}+\frac{2}{\mu_{P}}$$

(4) $$t_{mP}=\tau_{P}+\frac{P_{m}}{2v_{P}}$$

where, *P* is the product determined (*X*, *G*, *L*, or *A*); *t* is the time of culture (h); *P_m_* is the maximum product production (g/L for *X*, *L* and *A* and dimensionless for *G*); *v_P_* is the maximum production rate (g L^−1^, h^−1^ for *X*, *L* and *A* and h^−1^ for *G*); *λ_P_* is the products lag phase (h); *μ_P_* is the specific maximum production rate (h^−1^); *τ_P_* is the time required to achieve half of the maximum production (h) and *τ_mP_* is the time required to reach the maximum production (h). Moreover, the yields of bioproductions on soluble protein uptake (*Y_P_*/*Y_Pr_*) and reducing sugars (*Y_P_*/*Y_RS_*) consumption were determined.

### 2.5. Economical Evaluation of Bioproduction Costs

A simple and preliminary study of economical sustainability of the LAB bioproduction costs was also carried out. Taking as reference the market prices of the MRS ingredients and the values of *X_m_*, *G_m_*, *L_m_*, and *A_m_* compiled in Table 2 and Supplementary Materials Tables S1–S6, we quantified the cost of production of biomass (in €/g), cells (in €/cell), and lactic (in €/g) and acetic (in €/g) acids in each alternative media formulated with fish peptones and in the commercial MRS media. In these calculations, we have not incorporated the cost of production of marine peptones (energy and reagents). These costs are highly dependent on the production scale of peptones (relatively higher on a smaller level of production) and difficult to quantify on a laboratory scale. It is important to note that our strategy of head and skin bioconversion are complementary ways to the recovery of fish mince from fish discards biomasses that have to be landed under the LO normative.

### 2.6. Numerical Fittings and Statistical Analyses

Fitting procedures and parametric estimations calculated from the results were carried out by minimizing the sum of quadratic differences between the observed and model-predicted values, using the non-linear least-squares (quasi-Newton) method provided by the macro-‘Solver’ of the Microsoft Excel spreadsheet. Confidence intervals from the parametric estimates (Student’s *t* test) and consistence of mathematical models (Fisher’s F test) were evaluated by “SolverAid” macro (Levie’s Excellaneous website: http://www.bowdoin.edu/~rdelevie/excellaneous). The significance of comparisons between fish media and MRS were analyzed by ANOVA with a significance level of *p* < 0.05.

## 3. Results and Discussion

Under the LO framework, two scenarios for the management of fish discards must be taken into account when they are discharged in ports: (a) those species with legal size and subject to total allowed catches (TACs) which cannot be fully absorbed by markets and (b) those species below minimum legal size with or without TACs regulation. In the first case, it is possible to produce fish mince for human consumption, but heads, skins, and bones are generated as by-products. In the second case, other applications beyond direct human consumption must be explored [27]. This is the reason why, in the present work, we have developed a valuable bioprocessing alternative to valorize whole individuals and by-products for the ten most discarded species in European fishing boats (Figure 1).

The content of soluble protein and of reducing and total sugars of the marine peptones is summarized in Table 1. The protein value of peptones ranged from 28 to 54 g/L and depended on both species and substrate type. Thus, whole specimens as substrates, and megrim, blue whiting, grenadier, and Atlantic horse mackerel, among the fish species, showed the higher concentration of proteins. The concentration of total sugars was always lower than 1.6 g/L, with less than half of this figure in the form of reducing sugars, mainly glucose (<0.5 g/L).

The contribution of these sugars to the culture media for LAB growth was testimonial (as much as 0.5 g/L of TS and 0.15 g/L of RS), since their inclusion as an ingredient was added at the concentration of Pr = 10 g/L. The degree of hydrolysis of peptones varied from 11% to 47% and the average molecular weights of the peptides present were around 700–1500 Da (data not shown) [4,14,32].

### 3.1. Growth of LAB on Marine Peptones from Fish Discards

The selection of the LAB, *Lactobacillus brevis* (Lb 1), *Lactobacillus plantarum* (Lb 2), *Lactobacillus cassei* (Lb 3), and *Leuconostoc mesenteroides* (Ln), was motivated by its well-known technological characteristics, including its use as starters of meat, milk, and vegetable fermentations [34,35,36] and their probiotic ability in aquaculture growth, and human gut and respiratory diseases [37,38,39,40].

The kinetics of the bioproductions (biomass-X, viable cells-G, and lactic-L and acetic acid-A) for the case of Lb 1 in the 30 media with marine peptones, as well as in MRS control medium, are displayed in (Supplementary Material Figure S1). The time-course of pH and the consumptions of reducing sugars and soluble protein were also recorded but are not shown, as the present study focuses on growth and metabolite production. The pH-profiles were similar in all cultivations showing conventional decreasing logistic patterns with non-null asymptote [41]. The uptake of RS was almost exhaustive at the end of the cultures; however, Pr total consumptions were always lower than 2.4 g/L.

Experimental data of Lb 1 bioproductions were accurately described by Equation (1) including the complete description of kinetic phases derived from Equations (2)–(4). Table 2 summarizes the numerical values of the mentioned parameters. From a statistical viewpoint, the agreement between experimental and predicted data was excellent (R^2^ variations from 0.989 to 0.999, from 0.958 to 0.992, from 0.975 to 0.999 and from 0.944 to 0.998 for X, G, L and A, respectively). The consistency of fits was, in all situations, supported (*p* < 0.005 from F-Fisher test) and all parameters were statistically significant (for α = 0.05, *t*-Student test). Identical experimental profiles were also found in the growth of another LAB [26,32], yeasts [42], and marine bacteria [43] and in the production of microbial metabolites such as nisin [26], enzymes [44], and biopolymers [45]. These sigmoid patterns are commonly observed in the growth of organisms, including bacteria and mammalians, in batch and limiting nutrient conditions [4,33]. Additionally, the Equation (1) derived from an autocatalytic pseudo-mechanism [46] and reparametrized, as here shown, is a perfect tool to simulate and typify all the sigmoid growth phases of the organisms involved [33,47], defining a set of parameters of clear biological interest for comparative purposes.

Taking into account the numerical estimations, the higher values of maximum production of biomass (*X_m_*) was obtained in Wh_Bo and Wh_Ha and was nevertheless similar in most other media, including MRS. He_Gr and He_BW showed slightly inferior biomass production. The rate parameters for Lb 1 growth (*v_x_*, *μ_x_*) and the time-dependent coefficients (*λ_x_*, *τ_x_*, *t_mx_*) were almost always statistically similar in the different peptones evaluated.

As expected, the formation of viable cells followed similar results to those described for biomass: all peptones supported the maximum growth of cells (*G_m_*) in more identical productive terms than MRS. The rest of cell parameters summarized in Table 2 was also indistinguishable to those promoted by the control medium. The two main metabolites for a heterofermentative strain as Lb 1, lactic and acetic acid, were also analyzed in the postincubates of the fermentations. Lactic and acetic acids followed behaviors of primary and mixed metabolites (data not shown) respectively, according to the definition of Luedeking and Piret [48]. The maximum productions of lactic (*L_m_*) in He_Gr (14.5 g/L) and Sk_Ha (11.9 g/L) were the highest and the lowest from the peptones but, in statistical terms, significant differences were almost not observed (*p* > 0.05) between all media. Similar findings of a lack of statistical differences were observed, in most cases, for the lactic acid production rates and time-dependent parameters. The ranges of acetic acid concentration predicted (*A_m_*= 1.91–2.80 g/L) correlate with those previously reported in the fermentation of squid effluents [41]. The response here was not very far from what was shown in X, G, and L (Table 2).

**Figure 2:**
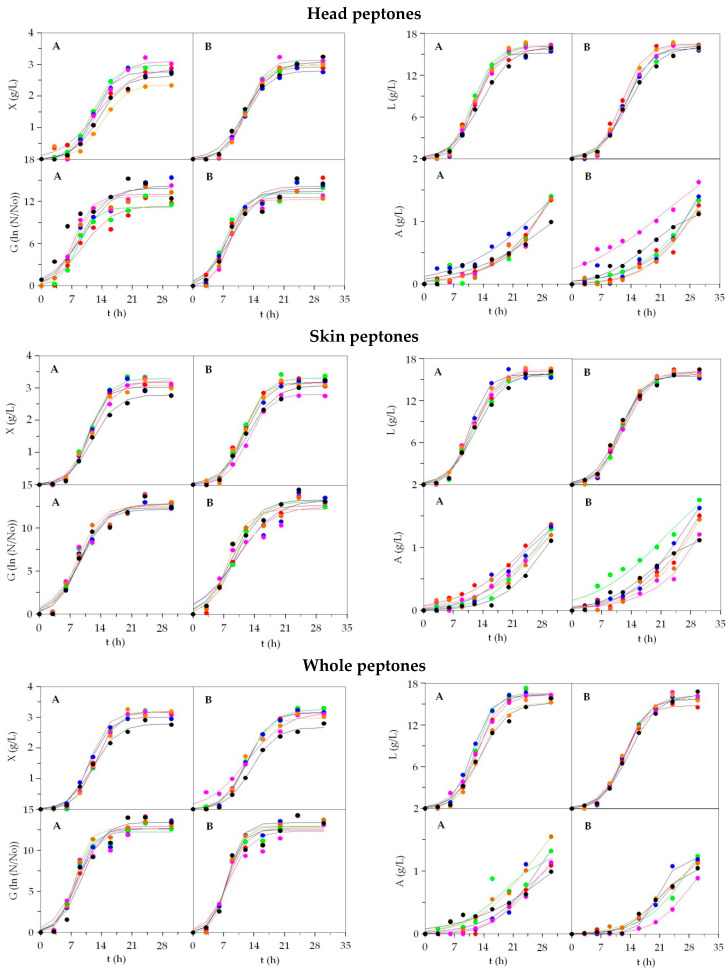
Culture kinetics of Lb 2 in alternative media based on marine peptones from discarded fish and by-products. Peptones A: ●: BW; ●: RS; ●: Ma; ●: Po; ●: Gu; ●: MRS. Peptones B: ●: Gr; ●: Me; ●: Ha; ●: Bo; ●: AHM; ●: MRS. Experimental data of biomass (X), viable cells (G), lactic acid (L), and acetic acid (A) were fitted to the logistic equation. The confidence intervals of experimental data (for two replicates) were in all cases less than 15% of the experimental mean values and omitted for clarity.

In Supplementary Material (Figure 2, Figures S2 and S3 and Tables S1–S6) we have included the results achieved for the fermentations of Lb 2, Ln, and Lb 3. Once again, the logistic equation was shown to be an excellent mathematical tool to model the bioproduction kinetics of Lb 2, Lb 3, and Ln: determination coefficients ranging 0.955–0.999, consistency and robustness of equations confirmed (*p*-values < 0.005).

For *L. plantarum* (Lb 2), the values of *X_m_* were numerically larger in media including Sk_peptones (ranging from 2.81–3.31 g/L) and Wh_peptones (from 2.98–3.27 g/L) than He_peptones from 2.37–3.10 g/L. Nevertheless, excluding some cases such as Sk_RS (higher) and He_Gu (lower), the differences between alternative media and control were practically non-existent (*p* > 0.05). The highest values were reached in Sk_Me, Sk_RS, and Wh_Bo. There were no differences either among media for the biomass rates and time-dependent parameters. A similar lack of significance, in the comparison between fermentations, was also observed for the production of viable cells and lactic acid (Supplementary Material, Tables S1 and S2). Thus, the exceptional capacity of our peptones to substitute the commercial peptones present in MRS was revealed. This statement was in line with [49], which reports that peptones from viscera of swordfish, ray, and shark supported the fermentation of Lb 2 in equal or better conditions than MRS. Besides that, effluents obtained from chemical and enzymatic deproteinization of squid pens, containing a large concentration of soluble protein and good balances of amino acids, proved to be an excellent source of peptones for the growth and metabolite production from Lb 2 [41]. The data of acetic acid formation varied in the interval of 1–2 g/L depending on the peptone employed (Figure 2), but the lack of asymptote in the experimental trends made it impossible to obtain significant values for the parameters of the logistic equation. That is why precise comparisons cannot be made for this bioproduction.

Regarding *L. mesenteroides* (Ln) fermentations (Supplementary Material, Figure S2), *X_m_*-parameters in MRS were in the same order of magnitude as those reported in fish peptone media, and less in three cases, Sk_Ma was statistically lower and Sk_Ha together with Sk_Gu were higher (*p* < 0.05) than the control medium (Supplementary Material, Tables S3 and S4). There were no notable differences between the fermentations for the set of other parameters derived from the logistic equation applied to the biomass data Equations (1)–(4). The growth in terms of cell parameters were similar, without significant numerical changes, in all kinetics (*p* > 0.05). For *L_m_*, only two peptones (Sk_BW and Sk_RS) yielded lower productions of lactic acid, and in the rest of fish media, the values of maximum lactic acid concentration were equal to commercial media. Numerically, almost 18 g/L of lactic acid were produced in Sk_Bo, Sk_Ha, and He_AHM. Finally, the results of acetic acid were more varied than in the previous bioproductions: He_Po, He_Gr, He_AHM, and MRS were lower (1.25–1.57 g/L) in comparison with the best cases Sk_Ma, He_RS, Wh_Ma, and Wh_Ha (2.34–2.46 g/L). Cost-effective media including peptones of Sk_Bo, Wh_Bo, and He_Po produced the best results of maximum production of biomass in dry weight (>3.6 g/L) for *L. casei* (Lb 3) (Supplementary Material Figure S3). Those values were slightly but statistically higher than those generated in MRS. Meanwhile, Sk_Gu, Sk_Me, and He_Ha led to lower biomasses (around 2.8–2.9 g/L). No clear tendencies were found for growth rate estimations. The production of viable cells was statistically equal in all media and significant differences were not found (*p* > 0.05). Identical findings were defined for the rest of the numerical coefficients of cell growth (Supplementary Material, Tables S5 and S6). Lactic acid maximum productions were also statistically similar in all cultures, although differences of 2 g/L were observed among Wh_Gr (16.1 ± 1.5 g/L) and Sk_Ha (18.7 ± 1.4 g/L). The values of production rates (*v_L_*, *μ_L_*) and the time-dependent coefficients (*λ_L_*, *τ_L_*, *t_mL_*) followed a similar behavior to those found in growth. Aspmo et al. [50] also reported the viability of peptones from cod viscera hydrolysates for the production of *L. casei* biomass in batch fermentations. As shown in Lb 2, the estimates of *A_m_* were not determined in some cases with enough significance to establish comparisons. The real experimental data of acetic acid were produced in the range of 0.82–1.60 g/L (Supplementary Material, Figure S3).

On the other hand, the greatest maximum biomass and lactic acid productions were found in the pairs Lb 1/Lb 3 and Ln/Lb 3, respectively. Lag phases of bioproductions (*λ_p_*) were shorter in Ln cultures (3.3–5.6 h) and longer in Lb 3 (6.9–10.0 h). The times needed to achieve the asymptotic phase of maximum bioproductions (*t_mP_*) were longer in Lb 1 fermentations (10–17 h). In relation to the production yields, we have summarized in (Supplementary Material Table S7) the minimum and maximum values of the ratios *Y_P/RS_* and *Y_P/Pr_* obtained for all bacteria and bioproductions. On the basis of these ranges, we can establish the following partial conclusions: (a) Lb 1 was the most efficient strain in the production of biomass and acetic acid, Ln showed the highest yields in the formation of cells, and Ln together with Lb 2 revealed the best results of lactic acid production in relation to the consumption of reducing sugars and proteins, respectively; (b) MRS and Sk_Me were the most consistent media showing maximum yields, and He_RS and Sk_RS the least effective peptones in various bioproductions.

The low and isolated differences in bioproductions between media could be due to the amino acid composition of peptones (Supplementary Material, Tables S8 and S9). For example, peptones from skins + bones presented higher levels of glycine and lower percentage of glutamic acid than heads and whole individuals. Nevertheless, the capacity of peptones was almost always similar or higher than that found in commercial meat extract and bactopeptone from MRS, proving that the fish peptones studied here present a good balance in the amino acid content, including those essential for many lactic acid bacteria (*L. helveticus*, *L. plantarum*, etc.), such as Ile, Leu, Cys, Glu, or Val [51,52,53].

LAB are microorganisms classified as fastidious from the point of view of the nutrient requirements for growth and metabolite production: they always need complex media including various inorganic salts, at least two peptones and yeast extract, one tensioactive and a source of sugar, with glucose being the most commonly employed [54,55,56,57]. Peptones as a source of organic nitrogen cannot be substituted by inorganic nitrogen and must contain a set of peptides of various sizes not completely replaceable by free amino acids [56,58]. This is the main reason, together with the technological/industrial importance of LAB, why these bacteria are a perfect biological entity to evaluate the potential use of materials from effluents and food waste as ingredients of culture media in order to: (1) reduce the production cost of LAB, (2) avoid or at least manage the environmental impact of waste, (3) increase sustainability in the food chain, including the optimal valorization of by-products. The present results have shown the validity of fish discarded as a source of marine peptones to support the growth and metabolic activities of *L. brevis*, *L. plantarum*, *L. casei* and *L. mesenteroides*. These highlights are in concordance with the validity found for other protein sources recovered from residues and wastewaters of marine origin [26,41,50]. In addition, these new outcomes complement the previous results obtained in the production of pediocin AH-1 in these marine peptones of species discarded from the fishing activities [4,14,32].

Wastes and discards from fishing and aquaculture activities have been extensively employed as a source of bioactive peptides [59,60], oils rich in omega-3 fatty acids [61], silages [17], mince [28], aquaculture feed [15], or fish protein hydrolysates-FPH [4,14]. All of them are excellent options to manage and extract value from those uncomfortable and polluting substrates [62]. In our multiple proposal various of those strategies are also included: oils, FPH, and even muscle for the production of mince (Figure 1). A complete mass balance of obtained peptones and bioproductions was compiled in this Figure 1. Taking into consideration the productive ranges—depending on the type of substrate, the fish species and the LAB evaluated—, starting from 1 kg of discarded fish up to 60 mL of oil, 65 g of LAB biomass, 10^18^ cfu and 234 g of lactic acid could be produced at most. When fish mince is incorporated as a valuable product to recover, the alternative protocol can also lead to the production of 40 mL, 16 g, 3 × 10^17^ cfu and 58 g of oil, LAB, viable cells, and lactic acid, respectively. These data are sufficiently important to be considered as an attractive process for the valorization of fish wastes.

### 3.2. Economical Evaluation of Low-Cost Media for LAB Bioproductions

We have determined that the cost of LAB bioproductions in all culture media studied on the basis of the prices of MRS components and the values of maximum bioproductions (*X_m_*, *G_m_*, *L_m_* and *A_m_*) from Table 2 and Tables S1–S6. In this context, we were able to estimate the reduction in costs driven by the substitution of commercial peptones (bactopeptone and meat extract) by fish discard peptones included in the alternative MRS media.

**Figure 3:**
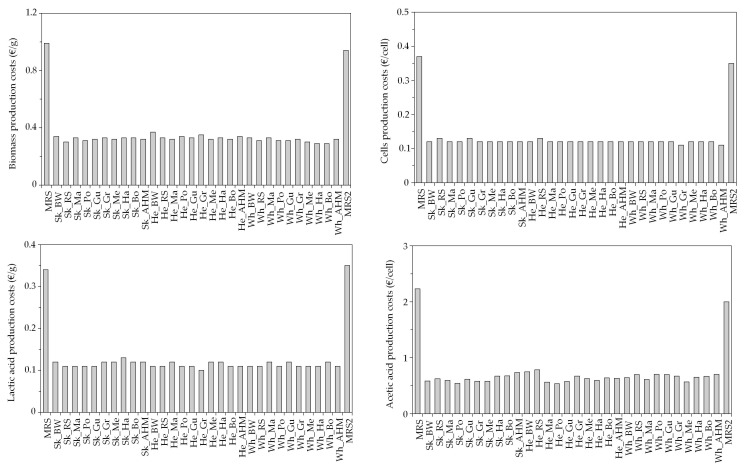
Economical evaluation of Lb 1 bioproduction costs in the culture media studied.

The cost of biomass and viable cell production for Lb 1 could be reduced at least three fold when marine peptones were present in alternative MRS media in comparison to commercial MRS. In a similar way, the production of lactic acid was three fold higher in commercial MRS than in fish peptone formulations and four times for the case of acetic acid (Figure 3). Similar findings were obtained for the rest of LAB employed in this work (Supplementary Material, Figures S4–S6). For certain peptones in the case of acetic acid, various histograms could not be displayed (Supplementary Material, Figures S4 and S6), due to the lack of significance of *A_m_* parameters (Supplementary Material, Tables S1, S2, S5 and S6). Nevertheless, the low concentration of acetic acid produced limits and discourages its interest as a bioproduction from an industrial point of view. These results of decreasing costs for LAB were in agreement with others previously reported that used effluents generated in the production of chitin from squid pens residues [41]. Although these calculations were established for the production of fish liquid peptones, an estimation of spray-drying dehydration of these peptones, to obtain powder-peptones, would reduce these mentioned benefits by at least 30%. In addition, the high concentrations of lactic acid and viable cell probiotics achieved and the low cost associated to their production may lead toward the establishment of a bio-based economy for the sustainable valorization of discarded fish in concordance with the suggestion commented by other authors for the bioconversion of organic wastes [63,64,65].

## 4. Conclusions

A sustainable biotechnological strategy to valorize fish discards was developed in the present work. Thirty marine peptones obtained from enzyme hydrolysis of fish discards (megrim, pouting, mackerel, etc.) were included in alternative complex media as organic nitrogen source (protein, peptides, and amino acids) for the growth and metabolic production of four lactic acid bacteria of technological properties (*L. brevis*, *L. casei*, *L. plantarum*, and *L. mesenteroides*). The results of biomass, cells, and lactic and acetic acids productions (using a kinetic approach) were always similar or higher than that provided by the MRS medium employed as control. The proposal presented in this work also led to a remarkable reduction of bioproduction costs, between 3–4 fold in comparison with those found in commercial MRS.

## Figures and Tables

**Table 1:** Basic biochemical composition of marine peptones (mean values ± confidence intervals). Pr: total soluble protein; RS: reducing sugars; TS: total sugars. Sk, He, and Wh mean skin, head, and whole individual. BW: blue whiting, RS: red scorpionfish, Ma: mackerel; Po: pouting; Gu: gurnard; Gr: grenadier; Me: megrim; Ha: hake; Bo: boardfish, and AHM: Atlantic horse mackerel.

Marine Peptones	Pr (g/L)	RS (g/L)	TS (g/L)	Marine Peptones	Pr (g/L)	RS (g/L)	TS (g/L)
**Sk_BW**	44.8 ± 3.1	0.13 ± 0.02	0.65 ± 0.06	**He_Gr**	29.4 ± 0.7	0.19 ± 0.05	0.83 ± 0.04
**Sk_RS**	39.7 ± 3.6	0.17 ± 0.04	0.74 ± 0.16	**He_Me**	34.5 ± 1.6	0.20 ± 0.02	0.62 ± 0.06
**Sk_Ma**	35.7 ± 2.4	0.42 ± 0.18	1.61 ± 0.50	**He_Ha**	29.5 ± 0.3	0.24 ± 0.09	0.79 ± 0.08
**Sk_Po**	42.7 ± 4.0	0.09 ± 0.01	0.45 ± 0.09	**He_Bo**	29.1 ± 4.8	0.16 ± 0.10	0.87 ± 0.12
**Sk_Gu**	39.7 ± 2.7	0.27 ± 0.03	0.92 ± 0.07	**He_AHM**	27.7 ± 0.9	0.36 ± 0.06	1.06 ± 0.07
**Sk_Gr**	42.2 ± 2.0	0.31 ± 0.04	0.73 ± 0.01	**Wh_BW**	47.8 ± 4.8	0.42 ± 0.09	1.20 ± 0.07
**Sk_Me**	40.4 ± 3.1	0.11 ± 0.09	0.50 ± 0.02	**Wh_RS**	36.8 ± 1.6	0.12 ± 0.00	0.60 ± 0.02
**Sk_Ha**	33.1 ± 0.5	0.13 ± 0.05	0.59 ± 0.02	**Wh_Ma**	36.4 ± 0.7	0.11 ± 0.00	0.74 ± 0.31
**Sk_Bo**	34.2 ± 0.5	0.45 ± 0.12	1.15 ± 0.05	**Wh_Po**	44.3 ± 2.3	0.14 ± 0.02	0.79 ± 0.05
**Sk_AHM**	38.4 ± 0.1	0.20 ± 0.03	0.70 ± 0.01	**Wh_Gu**	41.1 ± 5.4	0.24 ± 0.03	0.92 ± 0.00
**He_BW**	34.7 ± 3.4	0.31 ± 0.08	0.79 ± 0.01	**Wh_Gr**	47.1 ± 1.1	0.09 ± 0.01	0.50 ± 0.02
**He_RS**	39.2 ± 1.5	0.32 ± 0.09	1.25 ± 0.06	**Wh_Me**	53.9 ± 5.1	0.28 ± 0.12	1.06 ± 0.25
**He_Ma**	31.4 ± 2.8	0.25 ± 0.07	0.89 ± 0.04	**Wh_Ha**	36.5 ± 1.7	0.16 ± 0.02	0.72 ± 0.08
**He_Po**	39.6 ± 0.4	0.21 ± 0.07	0.68 ± 0.05	**Wh_Bo**	39.3 ± 1.9	0.41 ± 0.06	1.31 ± 0.37
**He_Gu**	33.5 ± 5.8	0.32 ± 0.06	1.45 ± 0.26	**Wh_AHM**	47.6 ± 3.2	0.33 ± 0.10	1.40 ± 0.23

**Table 2:** Numerical values and confidence intervals for parameters obtained from experimental data of *Lactobacillus brevis* (Lb 1) modelled by Equations (1)–(4). R^2^ is the determination coefficient among experimental and predicted data. MRS1 and MRS2 are used as control commercial media in the two set of experiments. Different letters in each row (as superscript) mean significant difference between fish peptone media and control media (*p* < 0.05).

	Sk_BW	Sk_RS	Sk_Ma	Sk_Po	Sk_Gu	Sk_Gr	Sk_Me	Sk_Ha	Sk_Bo	Sk_AHM	He_BW	He_RS	He_Ma	He_Po	He_Gu	MRS1
**Biomass (X)**																
***X_m_***	4.39 ± 0.23 ^A^	4.91 ± 0.31 ^B^	4.60 ± 0.18 ^B^	4.88 ± 0.21 ^B^	4.72 ± 0.31 ^B^	4.51 ± 0.16 ^A^	4.65 ± 0.23 ^B^	4.56 ± 0.31 ^B^	4.49 ± 0.18 ^B^	4.66 ± 0.21 ^B^	4.06 ± 0.31 ^A^	4.51 ± 0.16 ^B^	4.69 ± 0.17 ^B^	4.42 ± 0.10 ^A^	4.51 ± 0.23 ^B^	4.90 ± 0.23 ^B^
***v_m_***	0.54 ± 0.14 ^A^	0.58 ± 0.18 ^A^	0.52 ± 0.10 ^A^	0.56 ± 0.12 ^A^	0.59 ± 0.20 ^A^	0.50 ± 0.08 ^A^	0.66 ± 0.14 ^A^	0.68 ± 0.18 ^A^	0.71 ± 0.10 ^A^	0.67 ± 0.12 ^A^	0.77 ± 0.20 ^A^	0.49 ± 0.08 ^A^	0.83 ± 0.19 ^A^	0.70 ± 0.09 ^A^	0.75 ± 0.24 ^A^	0.73 ± 0.20 ^A^
***λ_X_***	6.43 ± 1.18 ^A^	6.63 ± 1.38 ^A^	6.32 ± 0.87 ^A^	6.82 ± 0.96 ^A^	6.80 ± 1.42 ^A^	6.50 ± 0.80 ^A^	7.18 ± 1.18 ^A^	7.23 ± 1.38 ^A^	7.37 ± 0.87 ^A^	7.14 ± 0.96 ^A^	7.65 ± 1.42 ^A^	5.40 ± 0.80 ^A^	6.80 ± 0.72 ^A^	6.80 ± 0.47 ^A^	6.31 ± 1.08 ^A^	6.56 ± 1.00 ^A^
***μ_X_***	0.49 ± 0.14 ^A^	0.47 ± 0.15 ^A^	0.45 ± 0.09 ^A^	0.46 ± 0.10 ^A^	0.50 ± 0.18 ^A^	0.44 ± 0.08 ^A^	0.57 ± 0.14 ^A^	0.60 ± 0.15 ^A^	0.63 ± 0.09 ^A^	0.58 ± 0.10 ^A^	0.75 ± 0.18 ^A^	0.43 ± 0.07 ^A^	0.70 ± 0.17 ^A^	0.63 ± 0.09 ^A^	0.66 ± 0.23 ^A^	0.59 ± 0.17 ^A^
***τ_X_***	10.5 ± 0.7 ^A^	10.9 ± 0.8 ^A^	10.7 ± 0.5 ^A^	11.2 ± 0.6 ^B^	10.8 ± 0.8 ^A^	11.1 ± 0.5 ^B^	10.7 ± 0.7 ^A^	10.6 ± 0.8 ^A^	10.5 ± 0.5 ^A^	10.6 ± 0.6 ^A^	10.3 ± 0.8 ^A^	10.1 ± 0.5 ^A^	9.64 ± 0.39 ^A^	9.95 ± 0.26 ^A^	9.32 ± 0.56 ^A^	9.94 ± 0.55 ^A^
***t_mX_***	14.6 ± 1.5 ^A^	15.1 ± 1.8 ^A^	15.1 ± 1.1 ^A^	15.5 ± 1.3 ^A^	14.8 ± 1.8 ^A^	15.3 ± 1.4 ^A^	14.2 ± 1.5 ^A^	14.0 ± 1.8 ^A^	13.7 ± 1.1 ^A^	14.0 ± 1.3 ^A^	13.0 ± 1.8 ^A^	14.7 ± 1.4 ^A^	12.5 ± 0.9 ^A^	13.1 ± 0.6 ^A^	12.3 ± 1.3 ^A^	13.3 ± 1.2 ^A^
***R^2^***	0.995	0.993	0.997	0.997	0.992	0.998	0.995	0.997	0.999	0.998	0.993	0.992	0.997	0.999	0.995	0.995
**Cells (G)**																
***G_m_***	12.3 ± 1.1 ^A^	11.9 ± 1.1 ^A^	12.4 ± 0.9 ^A^	12.5 ± 1.2 ^A^	11.9 ± 1.0 ^A^	12.6 ± 1.1 ^A^	12.3 ± 1.1 ^A^	12.4 ± 1.1 ^A^	12.5 ± 0.9 ^A^	12.6 ± 1.2 ^A^	12.1 ± 1.0 ^A^	11.9 ± 1.1 ^A^	12.1 ± 1.1 ^A^	12.5 ± 1.4 ^A^	13.0 ± 1.5 ^A^	13.0 ± 1.2 ^A^
***v_G_***	1.60 ± 0.77 ^A^	1.47 ± 0.70 ^A^	1.59 ± 0.65 ^A^	1.44 ± 0.68 ^A^	1.74 ± 0.87 ^A^	1.56 ± 0.71 ^A^	2.62 ± 0.78 ^A^	1.55 ± 0.70 ^A^	1.38 ± 0.65 ^A^	1.40 ± 0.68 ^A^	1.19 ± 0.87 ^A^	1.66 ± 0.71 ^A^	1.80 ± 0.92 ^A^	1.44 ± 0.84 ^A^	1.61 ± 0.99 ^A^	1.59 ± 0.79 ^A^
***λ_G_***	4.50 ± 2.07 ^A^	3.84 ± 2.17 ^A^	4.64 ± 1.77 ^A^	4.31 ± 2.29 ^A^	4.56 ± 1.96 ^A^	4.35 ± 2.06 ^A^	5.72 ± 2.07 ^A^	4.02 ± 2.17 ^A^	4.50 ± 1.77 ^A^	3.86 ± 2.29 ^A^	4.42 ± 1.96 ^A^	5.23 ± 2.06 ^A^	4.80 ± 1.97 ^A^	3.55 ± 2.80 ^A^	4.24 ± 2.78 ^A^	3.51 ± 2.28 ^A^
***μ_G_***	0.52 ± 0.26 ^A^	0.49 ± 0.25 ^A^	0.51 ± 0.22 ^A^	0.46 ± 0.23 ^A^	0.59 ± 0.31 ^A^	0.49 ± 0.24	0.86 ± 0.26 ^A^	0.50 ± 0.25 ^A^	0.44 ± 0.22 ^A^	0.45 ± 0.23 ^A^	0.39 ± 0.31 ^A^	0.56 ± 0.24 ^A^	0.59 ± 0.32 ^A^	0.46 ± 0.28 ^A^	0.49 ± 0.32 ^A^	0.49 ± 0.25 ^A^
***τ_G_***	8.35 ± 1.12 ^A^	7.90 ± 1.18 ^A^	8.54 ± 0.96 ^A^	8.66 ± 1.26 ^A^	8.00 ± 1.10 ^A^	8.40 ± 1.11 ^A^	8.06 ± 1.12 ^A^	8.02 ± 1.18 ^A^	9.04 ± 0.96 ^A^	8.36 ± 1.26 ^A^	9.52 ± 1.05 ^A^	8.81 ± 1.12 ^A^	8.18 ± 1.05 ^A^	7.89 ± 1.54 ^A^	8.29 ± 1.5 ^A^	7.62 ± 1.25 ^A^
***t_mG_***	12.2 ± 2.4 ^A^	12.0 ± 2.5 ^A^	12.4 ± 2.1 ^A^	13.0 ± 2.7 ^A^	11.4 ± 2.2 ^A^	12.4 ± 2.4 ^A^	10.4 ± 2.4 ^A^	12.0 ± 2.5 ^A^	13.6 ± 2.1 ^A^	12.8 ± 2.7 ^A^	14.6 ± 2.2 ^A^	12.4 ± 2.4 ^A^	11.6 ± 2.2 ^A^	12.2 ± 3.3 ^A^	12.3 ± 1.0 ^A^	11.7 ± 2.7 ^A^
***R^2^***	0.982	0.981	0.988	0.982	0.982	0.984	0.976	0.974	0.973	0.972	0.971	0.976	0.958	0.965	0.969	0.978
**Lactic acid (L)**																
***L_m_***	12.5 ± 1.1 ^A^	13.1 ± 0.7 ^A^	13.4 ± 0.4 ^A^	13.3 ± 0.8 ^A^	13.2 ± 0.6 ^A^	12.4 ± 0.6 ^A^	12.2 ± 1.1 ^A^	11.9 ± 0.6 ^A^	12.8 ± 0.4 ^A^	13.0 ± 0.8 ^A^	13.6 ± 0.6 ^A^	13.1 ± 0.5 ^A^	12.8 ± 0.6 ^A^	13.7 ± 1.1 ^A^	13.6 ± 0.9 ^A^	13.2 ± 0.8 ^A^
***v_L_***	1.60 ± 0.73 ^A^	1.52 ± 0.36 ^A^	1.50 ± 0.21 ^A^	1.43 ± 0.36 ^A^	1.31 ± 0.23 ^A^	1.47 ± 0.32 ^A^	1.82 ± 0.73 ^A^	1.80 ± 0.36 ^A^	1.71 ± 0.21 ^A^	1.45 ± 0.36 ^A^	1.33 ± 0.23 ^A^	1.31 ± 0.32 ^A^	1.70 ± 0.42 ^A^	1.55 ± 0.60 ^A^	1.61 ± 0.50 ^A^	1.49 ± 0.43 ^A^
***λ_L_***	7.08 ± 1.91 ^A^	6.52 ± 1.09 ^A^	6.46 ± 0.66 ^A^	6.37 ± 1.26 ^A^	5.76 ± 0.94 ^A^	6.53 ± 0.98 ^A^	7.38 ± 1.91 ^A^	7.37 ± 1.09 ^A^	6.85 ± 0.66 ^A^	6.45 ± 1.26 ^A^	6.17 ± 0.94 ^A^	6.29 ± 0.98 ^A^	6.32 ± 1.02 ^A^	6.05 ± 1.84 ^A^	5.52 ± 1.45 ^A^	5.61 ± 1.24 ^A^
***μ_L_***	0.51 ± 0.25 ^A^	0.47 ± 0.12 ^A^	0.45 ± 0.07 ^A^	0.43 ± 0.12 ^A^	0.39 ± 0.08 ^A^	0.47 ± 0.11 ^A^	0.60 ± 0.25 ^A^	0.60 ± 0.12 ^A^	0.54 ± 0.07 ^A^	0.45 ± 0.12 ^A^	0.39 ± 0.08 ^A^	0.40 ± 0.11 ^A^	0.53 ± 0.14 ^A^	0.45 ± 0.19 ^A^	0.47 ± 0.16 ^A^	0.45 ± 0.13 ^A^
***τ_L_***	11.0 ± 1.1 ^A^	10.8 ± 0.6 ^A^	11.0 ± 0.4 ^A^	11.0 ± 0.7 ^A^	10.8 ± 0.6 ^A^	10.8 ± 0.6 ^A^	10.7 ± 1.1 ^A^	10.7 ± 0.6 ^A^	10.6 ± 0.4 ^A^	10.9 ± 0.7 ^A^	11.3 ± 0.6 ^A^	11.3 ± 0.6 ^A^	10.1 ± 0.6 ^A^	10.5 ± 1.1 ^A^	9.76 ± 0.80 ^A^	10.1 ± 0.7 ^A^
***t_mL_***	14.9 ± 2.4 ^A^	15.1 ± 1.4 ^A^	15.4 ± 0.9 ^A^	15.7 ± 1.7 ^A^	15.9 ± 1.3 ^A^	15.0 ± 1.3 ^A^	14.1 ± 2.4 ^A^	14.0 ± 1.4 ^A^	14.3 ± 0.9 ^A^	15.4 ± 1.7 ^A^	16.4 ± 1.3 ^A^	16.3 ± 1.2 ^A^	13.8 ± 1.2 ^A^	14.9 ± 2.4 ^A^	14.0 ± 1.8 ^A^	14.5 ± 1.6 ^A^
***R^2^***	0.987	0.996	0.999	0.995	0.997	0.996	0.997	0.998	0.998	0.997	0.995	0.990	0.996	0.989	0.993	0.992
**Acetic acid (A)**																
***A_m_***	2.56 ± 0.33 ^A^	2.40 ± 0.41 ^A^	2.52 ± 0.18 ^A^	2.75 ± 0.33 ^B^	2.43 ± 0.22 ^A^	2.58 ± 0.16 ^B^	2.59 ± 0.28 ^A^	2.23 ± 0.16 ^A^	2.21 ± 0.10 ^A^	2.03 ± 0.23 ^A^	2.00 ± 0.15 ^A^	1.91 ± 0.43 ^A^	2.66 ± 0.20 ^B^	2.80 ± 0.66 ^A^	2.60 ± 0.21 ^B^	2.07 ± 0.29 ^A^
***v_A_***	0.33 ± 0.23 ^A^	0.28 ± 0.23 ^A^	0.20 ± 0.05 ^A^	0.18 ± 0.07 ^A^	0.20 ± 0.07 ^A^	0.19 ± 0.04 ^A^	0.16 ± 0.05 ^A^	0.16 ± 0.03 ^A^	0.15 ± 0.02 ^A^	0.15 ± 0.04 ^A^	0.18 ± 0.06 ^A^	0.14 ± 0.07 ^A^	0.13 ± 0.03 ^A^	0.15 ± 0.05 ^A^	0.17 ± 0.03 ^A^	0.15 ± 0.06 ^A^
***λ_A_***	5.77 ± 2.92 ^A^	5.19 ± 4.00 ^A^	3.42 ± 1.71 ^A^	3.00 ± 2.82 ^A^	3.40 ± 2.20 ^A^	2.89 ± 1.49 ^A^	2.06(NS)	7.38 ± 1.28 ^A^	6.21 ± 0.89 ^A^	7.50 ± 2.06 ^A^	8.28 ± 1.85 ^A^	8.46 ± 1.57 ^A^	4.39 ± 1.66 ^A^	7.72 ± 3.12 ^A^	7.31 ± 1.34 ^A^	6.87 ± 2.71 ^A^
***μ_A_***	0.52 ± 0.37 ^A^	0.46 ± 0.41 ^A^	0.32 ± 0.09 ^A^	0.27 ± 0.11 ^A^	0.34 ± 0.13 ^A^	0.30 ± 0.07 ^A^	0.25 ± 0.09 ^A^	0.28 ± 0.06 ^A^	0.28 ± 0.04 ^A^	0.30 ± 0.10 ^A^	0.36 ± 0.13 ^A^	0.28 ± 0.18 ^A^	0.30 ± 0.08 ^A^	0.21 ± 0.10 ^A^	0.25 ± 0.05 ^A^	0.30 ± 0.14 ^A^
***τ_A_***	9.65 ± 1.59 ^A^	9.53 ± 2.21 ^B^	9.61 ± 1.04 ^A^	10.5 ± 1.9 ^B^	9.37 ± 1.31 ^A^	9.59 ± 0.92 ^A^	10.1 ± 1.8 ^B^	14.6 ± 1.0 ^B^	13.5 ± 0.7 ^B^	14.2 ± 1.6 ^B^	13.8 ± 1.3 ^B^	15.5 ± 3.1 ^B^	11.2 ± 1.1 ^B^	17.2 ± 3.5 ^B^	15.2 ± 1.2 ^B^	13.6 ± 2.0 ^B^
***t_mA_***	13.5 ± 3.5 ^A^	13.9 ± 4.9 ^A^	15.8 ± 2.4 ^A^	18.0 ± 4.4 ^A^	15.3 ± 3.0 ^A^	16.3 ± 2.1 ^A^	18.2 ± 4.1 ^A^	21.8 ± 2.2 ^A^	20.7 ± 1.5 ^A^	20.9 ± 3.4 ^A^	19.3 ± 2.7 ^A^	22.6 ± 6.6 ^A^	17.9 ± 2.5 ^A^	26.7 ± 7.2 ^A^	23.1 ± 2.5 ^A^	20.3 ± 4.4 ^A^
***R^2^***	0.968	0.944	0.992	0.982	0.987	0.994	0.984	0.996	0.998	0.989	0.990	0.963	0.992	0.980	0.996	0.982
	**He_Gr**	**He_Bo**	**He_Ha**	**He_AHM**	**He_Me**	**Wh_BW**	**Wh_RS**	**Wh_Ma**	**Wh_Po**	**Wh_Gu**	**Wh_Gr**	**Wh_Bo**	**Wh_Ha**	**Wh_Me**	**Wh_AHM**	**MRS2**
**Biomass (X)**																
***X_m_***	4.26 ± 0.13 ^A^	4.65 ± 0.20 ^B^	4.55 ± 0.07 ^A^	4.40 ± 0.27 ^B^	4.61 ± 0.18 ^B^	4.59 ± 0.25 ^B^	4.89 ± 0.37 ^B^	4.50 ± 0.28 ^B^	4.80 ± 0.21 ^B^	4.88 ± 0.28 ^B^	4.75 ± 0.19 ^B^	5.08 ± 0.26 ^B^	5.08 ± 0.24 ^B^	4.92 ± 0.18 ^B^	4.62 ± 0.26 ^B^	4.89 ± 0.25 ^B^
***v_m_***	0.79 ± 0.17 ^A^	0.75 ± 0.19 ^A^	0.86 ± 0.10 ^B^	0.83 ± 0.34 ^A^	0.81 ± 0.21 ^A^	0.83 ± 0.28 ^A^	0.68 ± 0.28 ^A^	0.97 ± 0.48 ^A^	0.72 ± 0.18 ^A^	0.71 ± 0.23 ^A^	0.66 ± 0.15 ^A^	0.62 ± 0.17 ^A^	0.66 ± 0.16 ^A^	0.67 ± 0.13 ^A^	0.64 ± 0.20 ^A^	0.53 ± 0.12 ^A^
***λ_X_***	6.23 ± 0.64 ^A^	7.09 ± 0.87 ^A^	6.19 ± 0.35 ^A^	7.21 ± 1.16 ^A^	6.17 ± 0.83 ^A^	7.43 ± 1.04 ^A^	6.94 ± 1.60 ^A^	7.18 ± 1.18 ^A^	7.13 ± 0.91 ^A^	6.43 ± 1.22 ^A^	6.30 ± 0.88 ^A^	4.91 ± 1.22 ^A^	6.07 ± 1.04 ^A^	6.17 ± 0.79 ^A^	6.13 ± 1.23 ^A^	6.84 ± 1.09 ^A^
***μ_X_***	0.74 ± 0.16 ^A^	0.64 ± 0.17 ^B^	0.75 ± 0.09 ^A^	0.76 ± 0.32 ^B^	0.70 ± 0.19 ^A^	0.72 ± 0.25 ^B^	0.56 ± 0.24 ^B^	0.86 ± 0.44 ^B^	0.60 ± 0.16 ^B^	0.58 ± 0.20 ^B^	0.56 ± 0.13 ^B^	0.49 ± 0.14 ^B^	0.52 ± 0.13 ^B^	0.54 ± 0.11 ^B^	0.56 ± 0.18 ^B^	0.43 ± 0.10 ^B^
***τ_X_***	8.95 ± 0.31 ^A^	10.2 ± 0.5 ^A^	8.85 ± 0.17 ^A^	9.84 ± 0.65 ^A^	9.03 ± 0.41 ^A^	10.2 ± 0.6 ^B^	10.5 ± 0.9 ^B^	9.50 ± 0.61 ^A^	10.5 ± 0.5 ^B^	9.89 ± 0.66 ^A^	9.91 ± 0.48 ^A^	9.00 ± 0.66 ^A^	9.94 ± 0.57 ^A^	9.86 ± 0.43 ^A^	9.73 ± 0.66 ^A^	11.5 ± 0.7 ^B^
***t_mX_***	11.7 ± 0.7 ^A^	13.3 ± 1.0 ^A^	11.5 ± 0.4 ^A^	12.5 ± 1.4 ^A^	11.9 ± 0.9 ^A^	13.0 ± 1.2 ^A^	14.1 ± 1.9 ^B^	11.8 ± 1.5 ^A^	13.8 ± 1.1 ^B^	13.3 ± 1.4 ^B^	13.5 ± 1.1 ^A^	13.1 ± 1.4 ^A^	13.8 ± 1.3 ^B^	13.6 ± 0.9 ^A^	13.3 ± 1.5 ^B^	16.2 ± 1.5 ^B^
***R^2^***	0.998	0.996	0.999	0.992	0.997	0.994	0.989	0.991	0.996	0.994	0.997	0.994	0.996	0.997	0.994	0.996
**Cells (G)**																
***G_m_***	13.0 ± 1.1 ^A^	13.0 ± 1.1 ^A^	12.7 ± 1.0 ^A^	12.3 ± 1.3 ^A^	12.6 ± 0.9 ^A^	12.9 ± 1.7 ^A^	12.8 ± 1.2 ^A^	13.0 ± 1.3 ^A^	12.9 ± 1.0 ^A^	13.0 ± 1.1 ^A^	13.3 ± 0.9 ^A^	12.9 ± 1.3 ^A^	13.0 ± 1.0 ^A^	12.9 ± 1.2 ^A^	13.1 ± 0.7 ^A^	12.2 ± 1.2 ^A^
***v_G_***	1.49 ± 0.62 ^A^	1.32 ± 0.53 ^A^	1.78 ± 0.80 ^A^	1.47 ± 0.81 ^A^	1.83 ± 0.77 ^A^	1.50 ± 0.98 ^A^	1.41 ± 0.63 ^A^	1.32 ± 0.60 ^A^	1.42 ± 0.54 ^A^	1.33 ± 0.54 ^A^	1.39 ± 0.44 ^A^	1.49 ± 0.79 ^A^	1.74 ± 0.72 ^A^	1.73 ± 0.89 ^A^	1.63 ± 0.48 ^A^	1.76 ± 0.97 ^A^
***λ_G_***	4.08 ± 2.02 ^A^	3.93 ± 2.16 ^A^	4.40 ± 1.81 ^A^	4.00 ± 2.59 ^A^	4.21 ± 1.65 ^A^	4.22 ± 3.10 ^A^	3.72 ± 2.25 ^A^	3.45 ± 2.48 ^A^	3.74 ± 1.91 ^A^	3.30 ± 2.17 ^A^	3.08 ± 1.70 ^A^	3.80 ± 2.54 ^A^	3.58 ± 1.76 ^A^	4.02 ± 2.17 ^A^	3.71 ± 1.35 ^A^	4.89 ± 2.17 ^A^
***μ_G_***	0.46 ± 0.20 ^A^	0.41 ± 0.18 ^A^	0.56 ± 0.26 ^A^	0.48 ± 0.28 ^A^	0.58 ± 0.26 ^A^	0.47 ± 0.32 ^A^	0.44 ± 0.21 ^A^	0.40 ± 0.20 ^A^	0.44 ± 0.18 ^A^	0.41 ± 0.18 ^A^	0.42 ± 0.14 ^A^	0.46 ± 0.26 ^A^	0.53 ± 0.21 ^A^	0.54 ± 0.23 ^A^	0.50 ± 0.16 ^A^	0.58 ± 0.33 ^A^
***τ_G_***	8.44 ± 1.11 ^A^	8.84 ± 1.22 ^A^	7.96 ± 0.97 ^A^	8.19 ± 1.41 ^A^	7.66 ± 0.90 ^A^	8.50 ± 1.70 ^A^	8.27 ± 1.24 ^A^	8.41 ± 1.39 ^A^	8.28 ± 1.06 ^A^	8.18 ± 1.21 ^A^	7.87 ± 0.94 ^A^	8.12 ± 1.39 ^A^	7.32 ± 1.24 ^A^	7.75 ± 0.96 ^A^	7.74 ± 0.74 ^A^	8.35 ± 1.20 ^A^
***t_mG_***	12.8 ± 0.6 ^A^	13.8 ± 2.7 ^A^	11.5 ± 2.0 ^A^	12.4 ± 3.0 ^A^	11.1 ± 0.8 ^A^	12.8 ± 3.7 ^A^	12.8 ± 2.7 ^A^	13.4 ± 3.1 ^A^	12.8 ± 2.3 ^A^	13.1 ± 2.7 ^A^	12.6 ± 2.1 ^A^	12.4 ± 3.0 ^A^	11.1 ± 2.0 ^A^	11.5 ± 2.5 ^A^	11.8 ± 1.6 ^A^	11.8 ± 2.4 ^A^
***R^2^***	0.984	0.984	0.985	0.974	0.986	0.964	0.982	0.980	0.986	0.983	0.989	0.975	0.986	0.979	0.992	0.979
**Lactic acid (L)**																
***L_m_***	14.5 ± 1.0 ^A^	13.3 ± 0.6 ^A^	12.7 ± 1.0 ^A^	13.3 ± 1.1 ^A^	12.6 ± 0.9 ^A^	13.3 ± 0.9 ^A^	13.7 ± 1.0 ^A^	13.0 ± 0.9 ^A^	13.4 ± 1.0 ^A^	13.0 ± 0.5 ^A^	13.6 ± 0.7 ^A^	12.9 ± 1.3 ^A^	13.8 ± 0.7 ^A^	13.9 ± 1.0 ^A^	13.1 ± 0.7 ^A^	13.7 ± 0.9 ^A^
***v_L_***	1.81 ± 0.67 ^A^	1.64 ± 0.42 ^A^	1.79 ± 0.81 ^A^	1.63 ± 0.60 ^A^	1.83 ± 0.77 ^A^	1.47 ± 0.51 ^A^	1.72 ± 0.67 ^A^	1.50 ± 0.77 ^A^	1.49 ± 0.81 ^A^	1.84 ± 0.42 ^A^	1.74 ± 0.47 ^A^	1.49 ± 0.79 ^A^	1.78 ± 0.45 ^A^	1.63 ± 0.58 ^A^	1.63 ± 0.48 ^A^	1.39 ± 0.34 ^A^
***λ_L_***	5.36 ± 1.64 ^A^	6.21 ± 1.02 ^A^	4.42 ± 1.81 ^A^	6.20 ± 1.84 ^A^	4.21 ± 1.65 ^A^	5.36 ± 1.45 ^A^	5.75 ± 1.64 ^A^	5.14 ± 1.65 ^A^	5.72 ± 1.81 ^A^	6.53 ± 0.89 ^A^	6.41 ± 1.14 ^A^	3.80 ± 2.54 ^A^	5.97 ± 1.07 ^A^	5.35 ± 1.66 ^A^	3.71 ± 1.35 ^B^	6.78 ± 1.25 ^A^
***μ_L_***	0.50 ± 0.19 ^A^	0.49 ± 0.14 ^A^	0.57 ± 0.27 ^A^	0.49 ± 0.19 ^A^	0.58 ± 0.26 ^A^	0.44 ± 0.16 ^A^	0.50 ± 0.19 ^A^	0.46 ± 0.26 ^A^	0.45 ± 0.27 ^A^	0.57 ± 0.14 ^A^	0.51 ± 0.15 ^A^	0.46 ± 0.26 ^A^	0.51 ± 0.14 ^A^	0.47 ± 0.18 ^A^	0.50 ± 0.16 ^A^	0.41 ± 0.11 ^A^
***τ_L_***	9.39 ± 0.90 ^A^	10.3 ± 0.6 ^B^	7.96 ± 0.98 ^A^	10.3 ± 1.1 ^B^	7.66 ± 0.90 ^A^	9.90 ± 0.80 ^A^	9.73 ± 0.90 ^A^	9.49 ± 0.90 ^A^	10.2 ± 1.0 ^B^	10.1 ± 0.5 ^A^	10.3 ± 0.6 ^B^	8.12 ± 1.39 ^A^	9.86 ± 0.59 ^A^	9.62 ± 0.92 ^A^	7.74 ± 0.74 ^A^	11.7 ± 0.8 ^B^
***t_mL_***	13.4 ± 2.0 ^A^	14.3 ± 1.2 ^A^	11.4 ± 2.0 ^B^	14.3 ± 2.4 ^A^	11.1 ± 1.9 ^B^	14.4 ± 1.8 ^A^	13.7 ± 2.0 ^A^	13.8 ± 1.9 ^A^	14.7 ± 2.0 ^A^	13.6 ± 1.1 ^B^	14.2 ± 1.4 ^A^	12.4 ± 3.0 ^A^	13.7 ± 1.3 ^A^	13.9 ± 2.0 ^A^	11.8 ± 1.6 ^B^	16.6 ± 1.7 ^A^
***R^2^***	0.990	0.994	0.985	0.994	0.986	0.991	0.994	0.991	0.997	0.997	0.995	0.975	0.996	0.991	0.992	0.995
**Acetic acid (A)**																
***A_m_***	2.23 ± 0.24 ^A^	2.34 ± 0.21 ^A^	2.51 ± 0.49 ^A^	2.37 ± 0.45 ^A^	2.39 ± 0.47 ^A^	2.33 ± 0.28 ^A^	2.14 ± 0.26 ^A^	2.45 ± 0.46 ^A^	2.12 ± 0.18 ^A^	2.14 ± 0.26 ^A^	2.23 ± 0.12 ^A^	2.24 ± 0.27 ^A^	2.30 ± 0.12 ^A^	2.64 ± 0.37 ^A^	2.12 ± 0.18 ^A^	2.33 ± 0.21 ^A^
***v_A_***	0.20 ± 0.07 ^A^	0.18 ± 0.05 ^A^	0.17 ± 0.08 ^A^	0.15 ± 0.06 ^A^	0.15 ± 0.07 ^A^	0.16 ± 0.05 ^A^	0.18 ± 0.07 ^A^	0.16 ± 0.07 ^A^	0.21 ± 0.07 ^A^	0.18 ± 0.07 ^A^	0.19 ± 0.03 ^A^	0.17 ± 0.06 ^A^	0.27 ± 0.07 ^A^	0.16 ± 0.04 ^A^	0.21 ± 0.07 ^A^	0.21 ± 0.08 ^A^
***λ_A_***	7.65 ± 2.17 ^A^	5.81 ± 1.85 ^A^	5.15 ± 3.91 ^A^	7.22 ± 3.20 ^A^	5.34 ± 3.78 ^A^	7.21 ± 2.14 ^A^	7.50 ± 2.42 ^A^	5.25 ± 3.70 ^A^	6.19 ± 1.88 ^A^	7.50 ± 2.42 ^A^	8.95 ± 1.01 ^B^	5.61 ± 2.55 ^A^	5.83 ± 1.20 ^A^	7.42 ± 2.18 ^A^	6.19 ± 1.88 ^A^	3.70 ± 2.18 ^A^
***μ_A_***	0.36 ± 0.15 ^A^	0.32 ± 0.10 ^A^	0.26 ± 0.16 ^A^	0.25 ± 0.12 ^A^	0.25 ± 0.15 ^A^	0.28 ± 0.09 ^A^	0.34 ± 0.15 ^A^	0.26 ± 0.15 ^A^	0.40 ± 0.15 ^A^	0.34 ± 0.15 ^A^	0.34 ± 0.06 ^A^	0.31 ± 0.13 ^A^	0.48 ± 0.13 ^A^	0.24 ± 0.08 ^A^	0.40 ± 0.15 ^A^	0.37 ± 0.15 ^A^
***τ_A_***	13.3 ± 1.5 ^A^	12.1 ± 1.3 ^A^	12.8 ± 3.0 ^B^	15.2 ± 2.8 ^A^	13.2 ± 3.0 ^B^	14.5 ± 1.7 ^A^	13.3 ± 1.7 ^A^	13.0 ± 2.9 ^B^	11.3 ± 1.1 ^B^	13.3 ± 1.7 ^A^	14.8 ± 0.7 ^A^	12.2 ± 1.8 ^B^	10.0 ± 0.7 ^B^	15.9 ± 2.0 ^A^	11.3 ± 1.1 ^A^	9.19 ± 1.26 ^B^
***t_mA_***	18.9 ± 3.2 ^A^	18.5 ± 2.8 ^A^	20.3 ± 6.6 ^A^	23.2 ± 6.0 ^A^	21.1 ± 6.6 ^A^	21.8 ± 3.7 ^B^	19.2 ± 3.7 ^A^	20.7 ± 6.4 ^A^	16.3 ± 2.6 ^A^	19.2 ± 3.7 ^A^	20.7 ± 1.5 ^B^	18.7 ± 4.0 ^A^	14.2 ± 1.5 ^A^	24.4 ± 4.3 ^B^	16.3 ± 2.6 ^A^	14.7 ± 2.8 ^A^
***R^2^***	0.986	0.990	0.965	0.978	0.968	0.990	0.984	0.969	0.990	0.984	0.998	0.984	0.995	0.990	0.990	0.985

## Supplementary Materials

The following are available online at https://www.mdpi.com/2304-8158/9/9/1239/s1, Figure S1: Culture kinetics of Lb 1, Figure S2: Culture kinetics of Ln, Figure S3: Culture kinetics of Lb 3, Figure S4: Economical evaluation of Lb 2 bioproduction costs, Figure S5: Economical evaluation of Ln bioproduction costs, Figure S6: Economical evaluation of Lb 3 bioproduction costs, Table S1: Parameter kinetics of Lb 2 cultures, Table S2: Continuation of Table S2, Table S3: Parameter kinetics of Ln cultures, Table S4: Continuation of Table S3, Table S5: Parameter kinetics of Lb 3 cultures, Table S6: Continuation of Table S5, Table S7: Ranges of productive yields, Table S8: Amino acids content of fish discard peptones, Table S9: Continuation of Table S8.
