# Supplementary material for: Bioconversion of Fish Discards through the Production of Lactic Acid Bacteria and Metabolites: Sustainable Application of Fish Peptones in Nutritive Fermentation Media
Source: Foods. 2020 Sep 4;9(9):1239. doi: 10.3390/foods9091239 (PMC7554814; doi:10.3390/foods9091239)
Supplement: Supplementary file 1 [file foods-09-01239-s001.pdf]

SUPPLEMENTARY MATERIAL

Bioconversion of fish discards through the production of lactic acid bacteria and metabolites: Sustainable application of fish peptones in nutritive fermentation media.

José Antonio Vázquez[1,2*], Ana I. Durán[1,2], Araceli Menduíña[1,2], Margarita Nogueira[1,2], Ana María Gomes[3], Joana Antunes[3], Ana Cristina Freitas[3], Esther Dagá[4], Paula Dagá[4] and Jesus Valcarcel[1,2]

[1] Grupo de Biotecnología y Bioprocesos Marinos, Instituto de Investigaciones Marinas (IIM-CSIC), C/ Eduardo Cabello, 6, CP 36208, Vigo, Galicia – España. jvazquez@iim.csic.es (J.A.V.); anais@iim.csic.es (A.I.D.); araceli@iim.csic.es (A.M.); marga@iim.csic.es (M.N.); jvalcarcel@iim.csic.es (J.V.)

[2] Laboratorio de Reciclado y Valorización de Materiales Residuales (REVAL), Instituto de Investigaciones Marinas (IIM-CSIC), C/ Eduardo Cabello, 6, CP 36208, Vigo, Galicia – España;

[3] Universidade Católica Portuguesa, CBQF - Centro de Biotecnologia e Química Fina - Laboratório Associado, Escola Superior de Biotecnologia, Rua Diogo Botelho 1327, 4169-005 Porto, Portugal. amgomes@porto.ucp.pt (A.M.G.); jch.antunes@gmail.com (J.A.); afreitas@porto.ucp.pt (A.C.F.)

[4] Bialactis Biotech S.L., Grupo Zendal, Lugar a Relva, S/N, CP36410, O Porriño, Pontevedra, Galicia-España. esther@bialactis.com (E.D.); paula@bialactis.com (P.D.)

* Correspondence: jvazquez@iim.csic.es

Head peptones

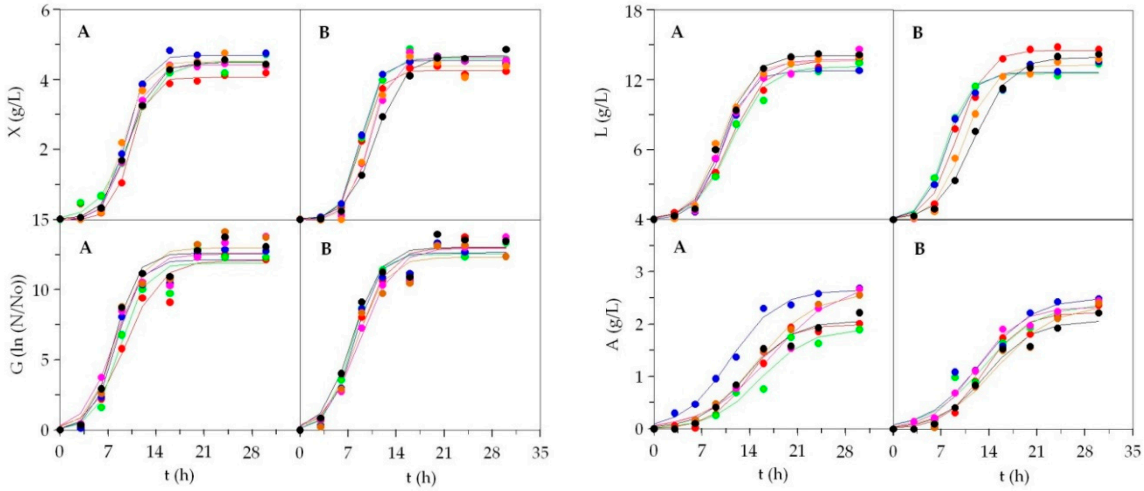

Skin peptones

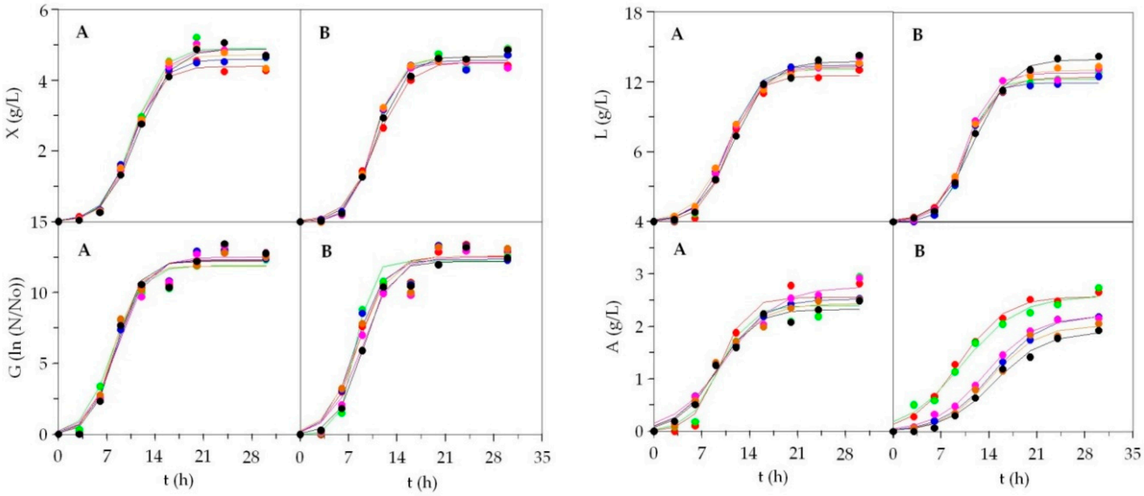

Whole peptones

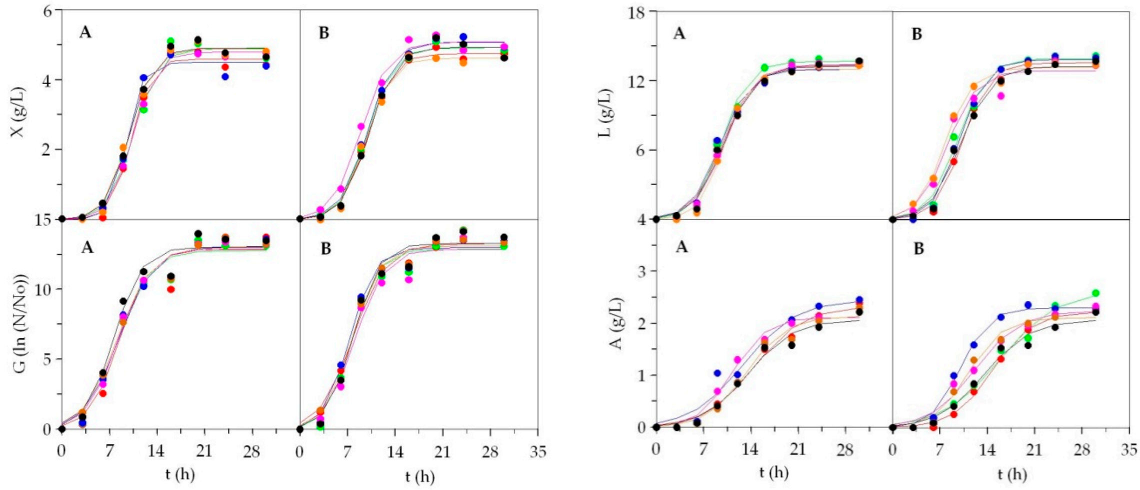

Figure S1. Culture kinetics of Lb 1 in alternative media based on marine peptones from discarded fish and by-products. Peptones A: ●: BW; ●: RS; ●: Ma; ●: Po; ●: Gu; ●: MRS. Peptones B: ●: Gr; ●: Me; ●: Ha; ●: Bo; ●: AHM; ●: MRS. Experimental data of biomass (X), viable cells (G), lactic acid (L) and acetic acid (A) were fitted to the logistic equation. The confidence intervals of experimental data (for two replicates) were in all cases lower than 15% of the experimental mean values and omitted for clarity.

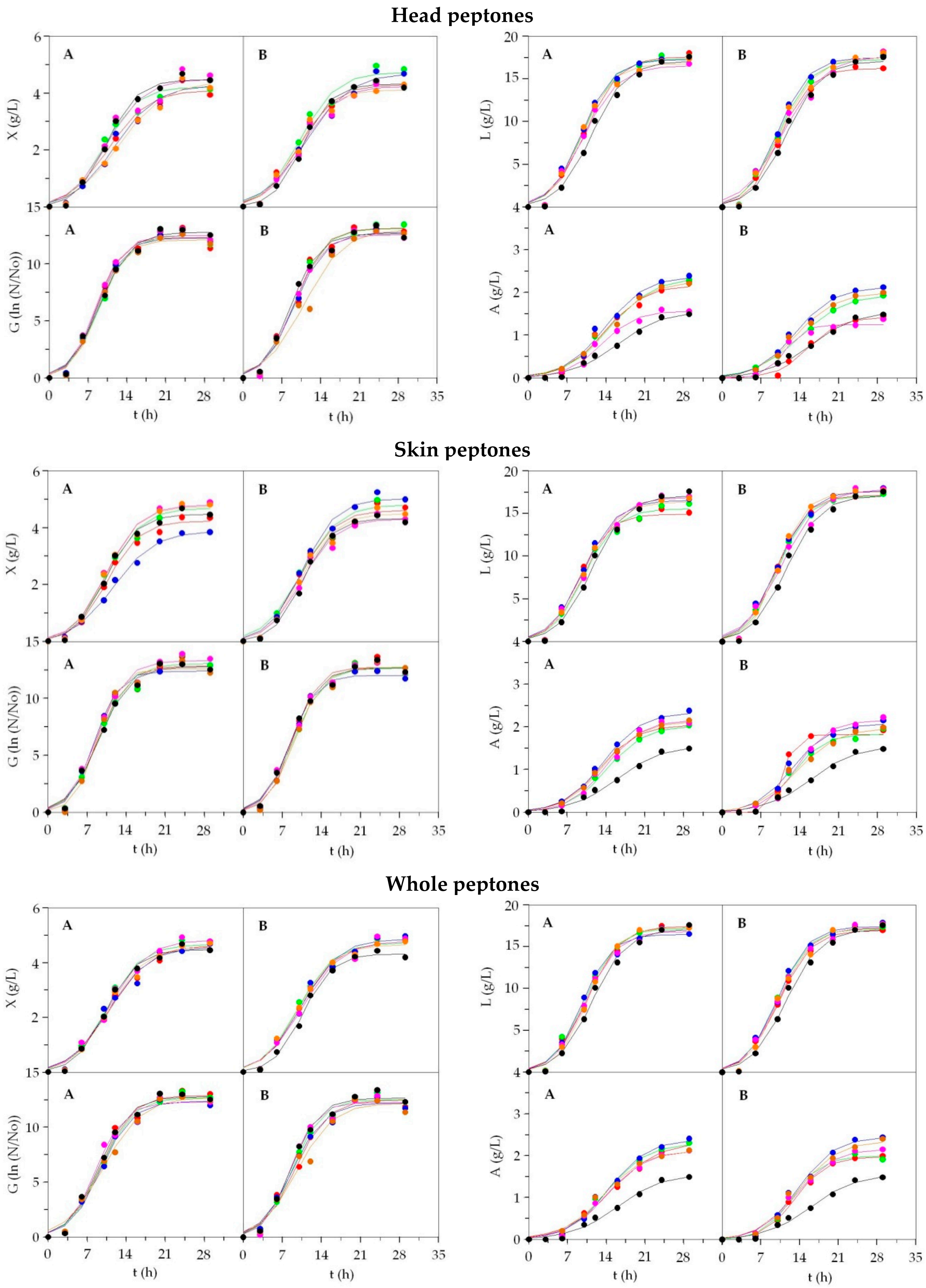

Figure S2. Culture kinetics of Ln in alternative media based on marine peptones from discarded fish and by-products. Peptones A: ●: BW; ●: RS; ●: Ma; ●: Po; ●: Gu; ●: MRS. Peptones B: ●: Gr; ●: Me; ●: Ha; ●: Bo; ●: AHM; ●: MRS. Experimental data of biomass (X), viable cells (G), lactic acid (L) and acetic acid (A) were fitted to the logistic equation. The confidence intervals of experimental data (for two replicates) were in all cases less than 15% of the experimental mean values and omitted for clarity.

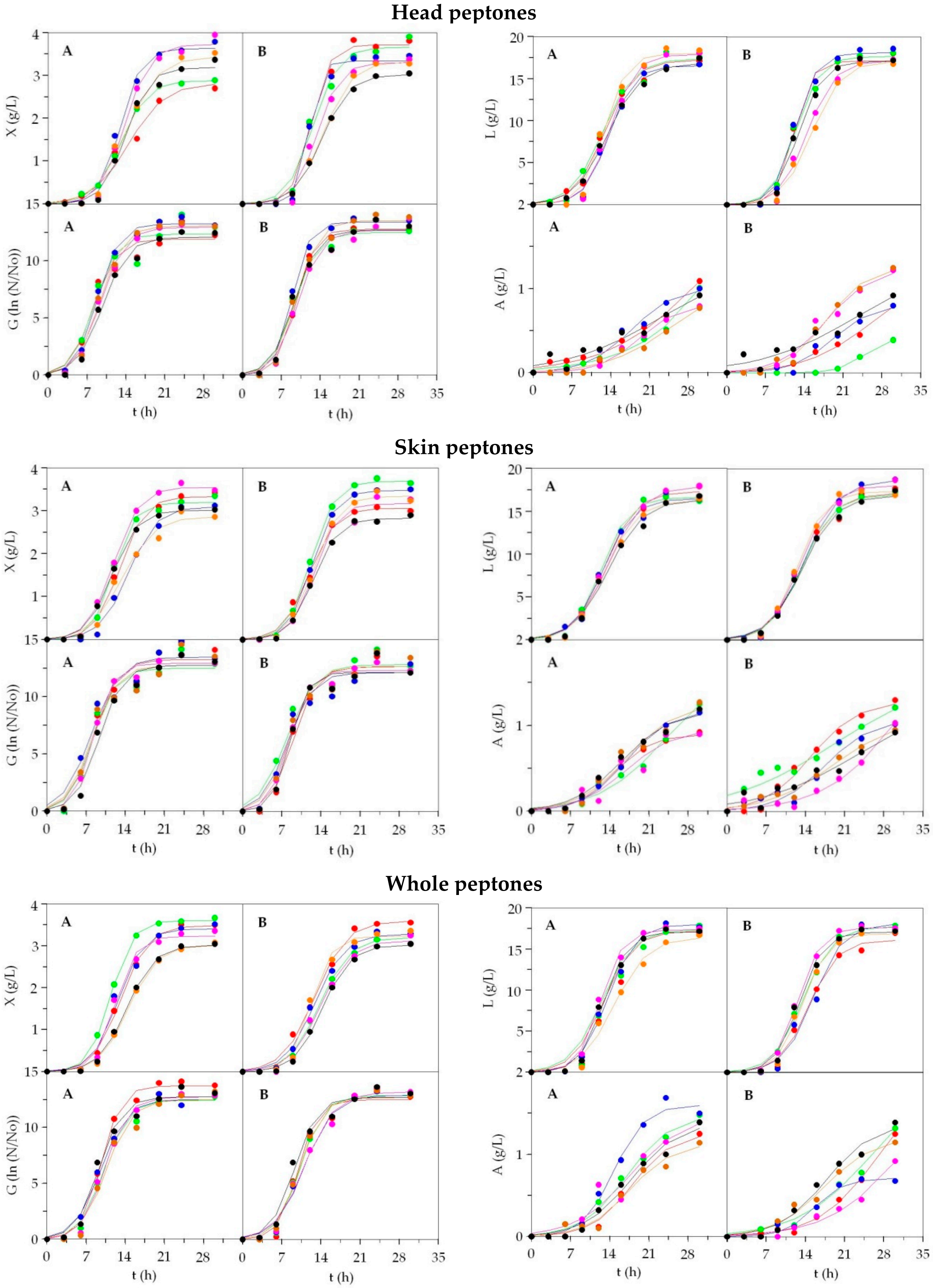

Figure S3. Culture kinetics of Lb 3 in alternative media based on marine peptones from discarded fish and by-products. Peptones A: ●: BW; ●: RS; ●: Ma; ●: Po; ●: Gu; ●: MRS. Peptones B: ●: Gr; ●: Me; ●: Ha; ●: Bo; ●: AHM; ●: MRS. Experimental data of biomass (X), viable cells (G), lactic acid (L) and acetic acid (A) were fitted to the logistic equation. The confidence intervals of experimental data (for two replicates) were in all cases less than 15% of the experimental mean values and omitted for clarity.

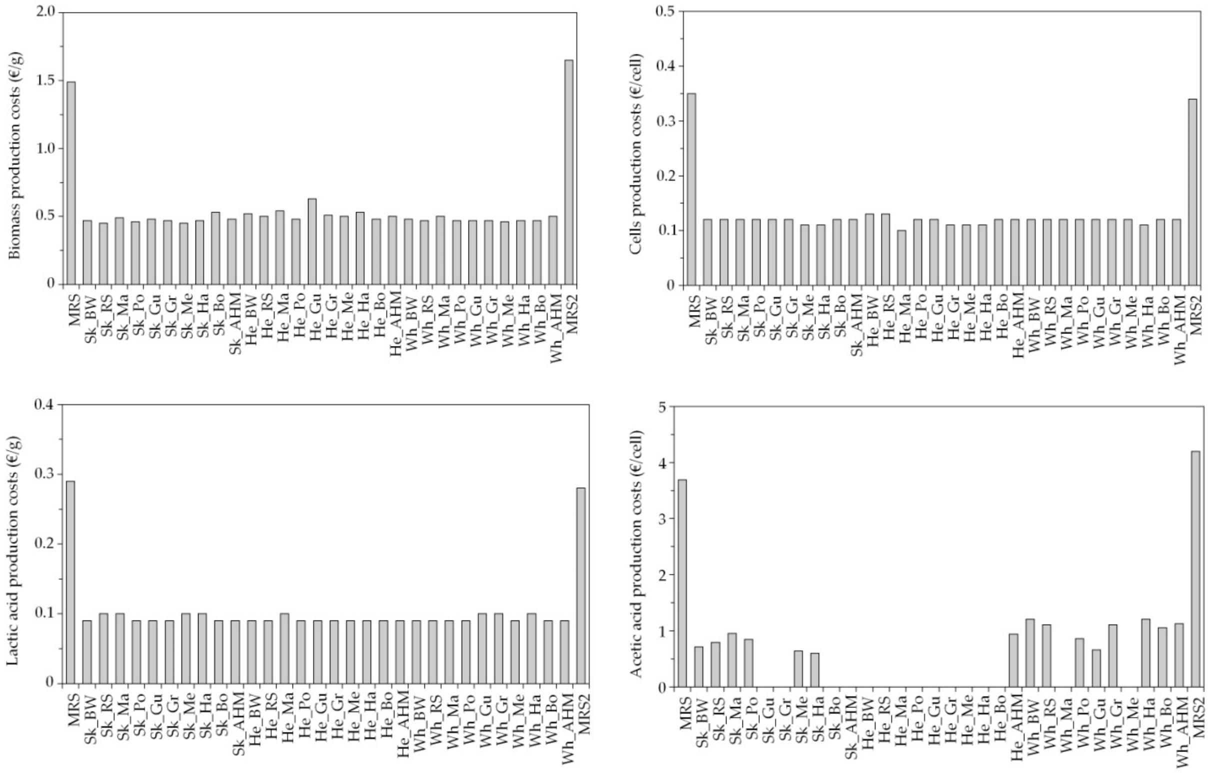

Figure S4. Economical evaluation of Lb 2 bioproduction costs in the culture media studied.

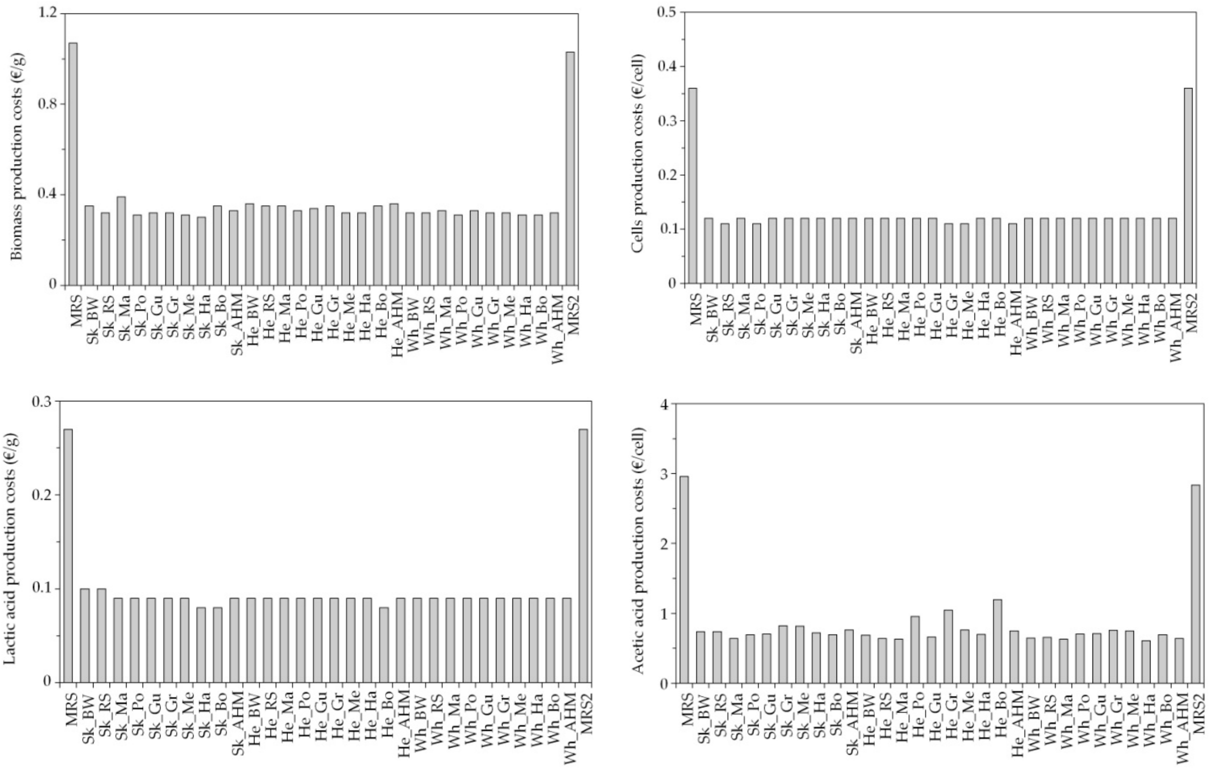

Figure S5. Economical evaluation of Ln bioproduction costs in the culture media studied.

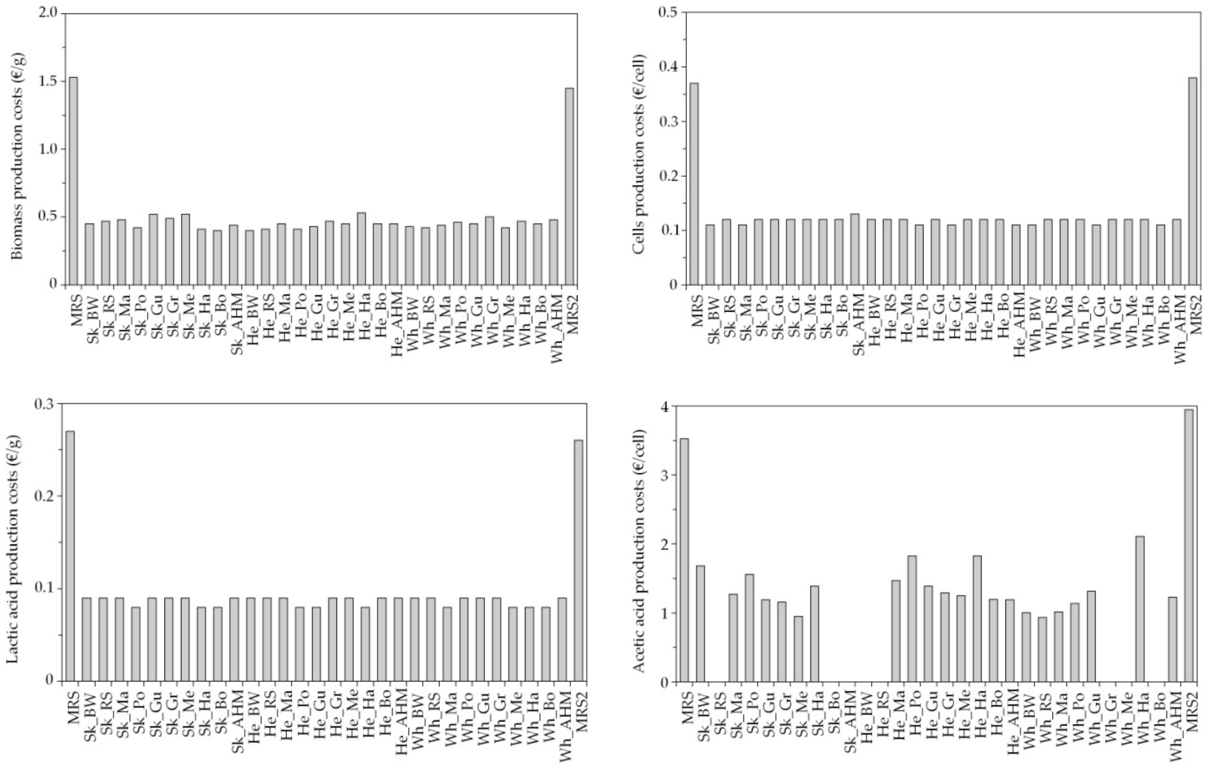

Figure S6. Economical evaluation of Lb 3 bioproduction costs in the culture media studied.

Table S1. Numerical values and confidence intervals for parameters obtained from experimental data of Lb 2 modelled by equations (1-4). R^2 is the determination coefficient among experimental and predicted data. NS: not significant. MRS1 and MRS2 are used as control commercial media. Different letters in each row (as superscript) means significant difference between fish peptone media and control media ($p < 0.05$).

	Sk_BW	Sk_RS	Sk_Ma	Sk_Po	Sk_Gu	Sk_Gr	Sk_Me	Sk_Ha	Sk_Bo	Sk_AHM	He_BW	He_RS	He_Ma	He_Po	He_Gu	MRS1
**Biomass (X)**																
X_m	3.17±0.20^{A}	3.29±0.19^{B}	3.03±0.27^{A}	3.23±0.21^{A}	3.09±0.20^{A}	3.19±0.20^{A}	3.31±0.23^{A}	3.19±0.16^{A}	2.81±0.12^{A}	3.15±0.25^{A}	2.87±0.34^{A}	2.98±0.06^{A}	2.77±0.17^{A}	3.10±0.25^{A}	2.37±0.30^{B}	2.89±0.20^{A}
v_m	0.38±0.12^{A}	0.36±0.09^{A}	0.40±0.19^{A}	0.29±0.07^{A}	0.31±0.08^{A}	0.33±0.09^{A}	0.34±0.10^{A}	0.31±0.06^{A}	0.29±0.05^{A}	0.33±0.11^{A}	0.18±0.05^{A}	0.30±0.03^{A}	0.31±0.08^{A}	0.30±0.08^{A}	0.21±0.09^{A}	0.25±0.07^{A}
λ_X	7.21±1.38^{A}	6.79±1.22^{A}	7.30±1.94^{A}	6.52±1.36^{A}	6.53±1.40^{A}	6.19±1.38^{A}	6.61±1.48^{A}	6.63±1.10^{A}	7.61±0.85^{A}	6.58±1.73^{A}	4.55±2.35^{A}	6.88±0.46^{A}	7.44±1.28^{A}	7.86±1.59^{A}	8.48±2.41^{A}	6.58±1.52^{A}
μ_X	0.48±0.16^{A}	0.44±0.12^{A}	0.52±0.26^{A}	0.36±0.09^{A}	0.40±0.12^{A}	0.42±0.12^{A}	0.41±0.13^{A}	0.39±0.09^{A}	0.41±0.08^{A}	0.42±0.16^{A}	0.25±0.08^{A}	0.40±0.04^{A}	0.44±0.13^{A}	0.38±0.12^{A}	0.36±0.17^{A}	0.36±0.11^{A}
τ_X	11.4±0.8^{A}	11.4±0.7^{A}	11.1±1.1^{A}	12.0±0.9^{A}	11.5±0.9^{A}	11.0±0.8^{A}	11.5±0.9^{A}	11.8±0.7^{A}	12.5±0.5^{A}	11.3±1.0^{A}	12.8±1.9^{A}	11.9±0.3^{A}	11.9±0.8^{A}	13.1±1.1^{A}	14.0±1.7^{A}	12.1±1.0^{A}
t_{mX}	15.6±1.8^{A}	15.9±1.6^{A}	15.0±2.4^{A}	17.6±2.0^{A}	16.5±2.0^{A}	15.8±1.9^{A}	16.3±2.0^{A}	16.9±1.6^{A}	17.3±1.2^{A}	16.1±2.4^{A}	20.9±4.2^{A}	16.9±0.6^{A}	16.5±1.8^{A}	18.4±2.3^{A}	19.5±3.6^{A}	17.6±2.2^{A}
R^2	0.993	0.995	0.986	0.995	0.994	0.994	0.993	0.997	0.998	0.990	0.986	0.999	0.995	0.993	0.983	0.994
**Cells (G)**																
G_m	12.8±1.6^{A}	12.8±1.9^{A}	12.1±1.2^{A}	12.8±2.0^{A}	12.5±1.4^{A}	12.6±1.1^{A}	13.2±1.4^{A}	13.4±2.9^{A}	12.5±2.6^{A}	12.2±1.4^{A}	11.4±2.1^{A}	11.2±1.4^{A}	14.3±2.5^{A}	13.0±1.5^{A}	12.7±1.2^{A}	12.3±1.2^{A}
v_G	1.03±0.47^{A}	1.00±0.53^{A}	1.13±0.50^{A}	0.96±0.53^{A}	1.27±0.66^{A}	1.18±0.71^{A}	1.08±0.43^{A}	0.76±0.44^{A}	0.78±0.49^{A}	1.38±0.79^{A}	0.84±0.54^{A}	1.38±0.87^{A}	0.95±0.51^{A}	1.48±0.88^{A}	1.45±0.71^{A}	1.23±0.97^{A}
λ_G	3.36±3.00^{A}	3.06 (NS)	3.71±2.55^{A}	2.54 (NS)	3.73±2.83^{A}	4.00±2.06^{A}	3.35±2.58^{A}	1.43 (NS)	1.45 (NS)	3.58±2.81^{A}	2.94 (NS)	4.36±2.86^{A}	2.04 (NS)	3.15±2.92^{A}	3.50±2.40^{A}	4.14±2.17^{A}
μ_G	0.32±0.26^{A}	0.31±0.25^{A}	0.37±0.22^{A}	0.30±0.23^{A}	0.41±0.31^{A}	0.38±0.24^{A}	0.33±0.15^{A}	0.23 (NS)	0.25±0.18^{A}	0.46±0.28^{A}	0.30±0.21^{A}	0.49±0.33^{A}	0.26±0.17^{A}	0.46±0.29^{A}	0.46±0.24^{A}	0.40±0.33^{A}
τ_G	9.60±1.12^{A}	9.44±1.18^{A}	9.08±0.96^{A}	9.23±1.26^{A}	8.66±1.05^{A}	9.34±1.12^{A}	9.43±1.55^{A}	10.2±1.2^{A}	9.43±3.39^{A}	7.98±1.54^{A}	9.70±2.86^{A}	8.41±1.56^{A}	9.61±2.79^{A}	7.54±1.60^{A}	7.88±1.32^{A}	9.14±1.15^{A}
t_{mG}	15.8±2.1^{A}	15.8±2.5^{A}	14.4±2.1^{A}	15.9±2.7^{A}	13.6±2.2^{A}	14.7±2.4^{A}	15.5±2.4^{A}	18.9±8.6^{A}	17.4±8.0^{A}	12.4±3.3^{A}	16.5±6.6^{A}	12.5±3.4^{A}	17.2±6.5^{A}	11.9±3.5^{A}	12.3±2.8^{A}	14.1±2.4^{A}
R^2	0.977	0.966	0.981	0.962	0.973	0.980	0.981	0.945	0.942	0.969	0.950	0.968	0.958	0.965	0.977	0.977
**Lactic acid (L)**																
L_m	15.9±1.1^{A}	15.6±1.2^{A}	15.7±0.9^{A}	16.3±1.2^{A}	16.6±0.9^{A}	16.2±0.8^{A}	15.5±0.6^{A}	15.6±0.9^{A}	16.2±0.9^{A}	15.9±0.8^{A}	15.8±1.1^{A}	16.2±0.6^{A}	15.2±0.8^{A}	16.2±1.0^{A}	16.3±1.1^{A}	15.9±1.4^{A}
v_L	1.48±0.38^{A}	1.39±0.41^{A}	1.84±0.53^{A}	1.43±0.38^{A}	1.45±0.27^{A}	1.29±0.22^{A}	1.64±0.29^{A}	1.56±0.36^{A}	1.44±0.29^{A}	1.53±0.30^{A}	1.38±0.35^{A}	1.60±0.25^{A}	1.77±0.45^{A}	1.50±0.34^{A}	1.56±0.39^{A}	1.23±0.33^{A}
λ_L	6.45±1.43^{A}	6.13±1.72^{A}	6.57±1.29^{A}	5.93±1.60^{A}	5.94±1.12^{A}	5.99±1.10^{A}	6.91±0.88^{A}	6.39±1.22^{A}	6.34±1.19^{A}	6.09±1.06^{A}	6.28±1.51^{A}	6.45±0.82^{A}	7.49±1.14^{A}	6.92±1.27^{A}	6.76±1.38^{A}	5.97±1.83^{A}
μ_L	0.37±0.11^{A}	0.36±0.12^{A}	0.47±0.14^{A}	0.35±0.11^{A}	0.35±0.07^{A}	0.32±0.06^{A}	0.42±0.08^{A}	0.40±0.10^{A}	0.35±0.08^{A}	0.38±0.08^{A}	0.35±0.10^{A}	0.40±0.07^{A}	0.47±0.13^{A}	0.37±0.09^{A}	0.38±0.11^{A}	0.31±0.10^{A}
τ_L	11.8±0.9^{A}	11.8±1.1^{A}	10.8±0.7^{A}	11.6±1.0^{A}	11.7±0.7^{A}	12.3±0.8^{A}	11.7±0.5^{A}	11.4±0.7^{A}	12.0±0.8^{A}	11.3±0.7^{A}	12.0±1.0^{A}	11.5±0.5^{A}	11.8±0.7^{A}	12.3±0.8^{A}	12.0±0.9^{A}	12.4±1.3^{A}
t_{mL}	17.2±2.1^{A}	17.4±2.5^{A}	15.1±1.7^{A}	17.3±2.3^{A}	17.4±1.6^{A}	18.5±1.7^{A}	16.4±1.2^{A}	16.4±1.7^{A}	17.6±1.7^{A}	16.5±1.5^{A}	17.8±2.2^{A}	16.6±1.1^{A}	16.1±1.5^{A}	17.7±1.9^{A}	17.2±2.0^{A}	18.9±2.8^{A}
R^2	0.995	0.992	0.994	0.993	0.997	0.997	0.998	0.996	0.996	0.997	0.994	0.998	0.996	0.956	0.995	0.992
**Acetic acid (A)**																
A_m	2.10±1.34	1.89±0.71	1.57±0.77	1.77±0.95	3.95 (NS)	5.68 (NS)	2.35±2.00^{A}	2.49±0.81^{A}	30.7 (NS)	4.40 (NS)	3.48 (NS)	55.9 (NS)	2.36 (NS)	6.93 (NS)	2.53 (NS)	20.3 (NS)
v_A	0.07±0.02^{A}	0.09±0.02^{A}	0.08±0.03^{A}	0.07±0.02^{A}	0.11 (NS)	0.17 (NS)	0.07±0.03^{A}	0.10±0.02^{A}	0.92 (NS)	0.15 (NS)	0.11 (NS)	1.59 (NS)	0.06±0.04^{A}	0.21 (NS)	0.10±0.07^{A}	0.10±0.05^{A}
λ_A	9.62±5.02^{A}	15.1±2.1^{A}	11.6±3.7^{A}	11.9±3.7^{A}	19.4 (NS)	21.7 (NS)	5.12 (NS)	13.7±2.1^{A}	4.01 (NS)	20.6 (NS)	18.2±12.7^{A}	44.6 (NS)	8.52 (NS)	25.2 (NS)	15.9±8.7^{A}	18.4±6.0^{A}
μ_A	0.13±0.06^{A}	0.19±0.06^{A}	0.19±0.12^{A}	0.15±0.10^{A}	0.11 (NS)	0.12±0.08^{A}	0.12±0.09^{A}	0.16±0.04^{A}	0.12±0.09^{A}	0.14±0.11^{A}	0.13±0.05^{A}	0.11 (NS)	0.11±0.06^{A}	0.12±0.10^{A}	0.15±0.09^{A}	0.19±0.15^{A}
τ_A	25.0±10.8^{A}	25.8±4.7^{A}	21.9±6.6^{A}	25.1 (NS)	37.2±1.3^{A}	40.8 (NS)	21.9±16.4	26.0±4.4^{A}	57.8 (NS)	35.4 (NS)	33.6±18.6^{A}	65.3 (NS)	27.3±20.9^{A}	41.5 (NS)	29.2±15.6^{A}	29.0±10.1^{A}
t_{mA}	40.3±17.3^{A}	36.5±7.8^{A}	32.3±12.5^{A}	38.3±13.0^{A}	55.0 (NS)	81.8 (NS)	38.7±28.2^{A}	38.2±7.2^{A}	76.4 (NS)	50.3 (NS)	49.0±24.7^{A}	77.8 (NS)	46.0±31.7^{A}	57.6 (NS)	42.5±22.9^{A}	39.7±15.1^{A}
R^2	0.982	0.994	0.970	0.987	0.957	0.974	0.952	0.996	0.976	0.969	0.991	0.953	0.971	0.967	0.981	0.990

Table S2. Continuation of Table S1.

	He_Gr	He_Bo	He_Ha	He_AHM	He_Me	Wh_BW	Wh_RS	Wh_Ma	Wh_Po	Wh_Gu	Wh_Gr	Wh_Bo	Wh_Ha	Wh_Me	Wh_AHM	MRS2
**Biomass (X)**																
X_m	2.93±0.21^{A}	2.99±0.13^{A}	2.80±0.15^{A}	3.14±0.20^{A}	2.99±0.09^{A}	3.10±0.11^{A}	3.20±0.13^{A}	3.01±0.09^{A}	3.18±0.11^{A}	3.20±0.20^{A}	3.16±0.14^{A}	3.27±0.20^{A}	3.17±0.16^{A}	3.19±0.37^{A}	2.98±0.28^{A}	3.09±0.30^{A}
v_m	0.30±0.09^{A}	0.32±0.06^{A}	0.27±0.06^{A}	0.35±0.10^{A}	0.32±0.04^{A}	0.35±0.05^{A}	0.35±0.06^{A}	0.33±0.05^{A}	0.34±0.05^{A}	0.34±0.09^{A}	0.31±0.05^{A}	0.29±0.06^{A}	0.30±0.06^{A}	0.18±0.05^{A}	0.30±0.11^{A}	0.25±0.08^{A}
λ_X	7.03±1.50^{A}	7.38±0.89^{A}	7.01±1.14^{A}	7.96±1.27^{A}	7.85±0.62^{A}	7.86±0.70^{A}	8.17±0.79^{A}	6.78±0.67^{A}	8.00±0.70^{A}	8.07±1.28^{A}	7.53±0.88^{A}	7.04±1.25^{A}	7.19±1.02^{A}	3.61±2.26^{A}	6.01±2.02^{A}	6.06±2.06^{A}
μ_X	0.40±0.15^{A}	0.43±0.09^{A}	0.38±0.09^{A}	0.45±0.13^{A}	0.43±0.06^{A}	0.45±0.07^{A}	0.44±0.08^{A}	0.44±0.07^{A}	0.43±0.07^{A}	0.42±0.12^{A}	0.40±0.07^{A}	0.35±0.08^{A}	0.38±0.08^{A}	0.23±0.18^{A}	0.40±0.17^{A}	0.32±0.11^{A}
τ_X	12.0±0.9^{A}	12.0±0.5^{A}	12.2±0.7^{A}	12.4±0.8^{A}	12.5±0.4^{A}	12.3±0.4^{A}	12.8±0.5^{A}	11.3±0.4^{A}	12.7±0.5^{A}	12.8±0.8^{A}	12.6±0.6^{A}	12.7±0.9^{A}	12.5±0.7^{A}	12.5±0.8^{A}	11.9±1.3^{A}	12.3±1.4^{A}
t_{mX}	17.0±2.1^{A}	16.6±1.2^{A}	17.4±1.6^{A}	16.9±1.8^{A}	17.1±0.9^{A}	16.7±1.0^{A}	17.3±1.1^{A}	15.8±0.9^{A}	17.4±1.0^{A}	17.6±1.8^{A}	17.6±1.3^{A}	18.4±1.9^{A}	17.7±1.5^{A}	21.4±4.2^{A}	17.0±2.9^{A}	18.5±3.1^{A}
R^2	0.993	0.998	0.996	0.995	0.999	0.999	0.998	0.999	0.999	0.995	0.998	0.996	0.997	0.988	0.992	0.990
**Cells (G)**																
G_m	14.2±1.7^{A}	13.2±1.4^{A}	13.5±1.4^{A}	12.6±1.0^{A}	12.3±1.0^{A}	13.0±1.3^{A}	12.3±1.0^{A}	12.6±1.6^{A}	12.7±2.1^{A}	12.7±0.8^{A}	12.8±1.5^{A}	12.9±1.3^{A}	13.4±1.0^{A}	12.4±2.0^{A}	13.0±1.3^{A}	13.3±1.6^{A}
v_G	1.26±0.65^{A}	1.50±0.83^{A}	1.46±0.72^{A}	1.69±0.71^{A}	1.66±0.78^{A}	1.33±0.61^{A}	1.62±0.76^{A}	1.49±0.93^{A}	1.16±0.82^{A}	1.91±0.67^{A}	1.51±0.88^{A}	1.81±1.00^{A}	1.93±0.80^{A}	1.26±0.95^{A}	2.19±1.40^{A}	1.10±0.52^{A}
λ_G	2.75(NS)	3.07±2.73	3.46±2.53	4.88±1.76	4.61±1.95	3.86±2.47	4.05±2.00	4.12±2.96	2.80(NS)	4.56±1.33	3.85±2.76	4.42±2.24	4.59±1.63	3.30(NS)	5.03±2.21	2.98(NS)
μ_G	0.36±0.20^{A}	0.46±0.27^{A}	0.44±0.23^{A}	0.54±0.24^{A}	0.54±0.27^{A}	0.41±0.20^{A}	0.53±0.26^{A}	0.47±0.31^{A}	0.37±0.28^{A}	0.60±0.22^{A}	0.47±0.29^{A}	0.56±0.32^{A}	0.58±0.25^{A}	0.41±0.33^{A}	0.68±0.45^{A}	0.33±0.17^{A}
τ_G	8.37±1.81^{A}	7.47±1.50^{A}	8.06±1.40^{A}	8.60±0.94^{A}	8.31±1.05^{A}	8.75±1.38^{A}	7.83±1.09^{A}	8.35±1.62^{A}	8.24±2.37^{A}	7.88±0.71^{A}	8.08±1.51^{A}	8.00±1.21^{A}	8.05±0.87^{A}	8.21±2.28^{A}	7.88±1.18^{A}	9.00±1.80^{A}
t_{mG}	14.0±4.1^{A}	11.9±3.2^{A}	12.7±3.0^{A}	12.3±2.0^{A}	12.0±2.2^{A}	13.6±3.1^{A}	11.6±2.3^{A}	12.6±3.5^{A}	13.7±5.3^{A}	11.2±1.5^{A}	12.3±3.2^{A}	11.6±2.5^{A}	11.5±1.8^{A}	13.1±5.0^{A}	11.0±2.4^{A}	15.0±4.1^{A}
R^2	0.968	0.970	0.977	0.987	0.984	0.980	0.983	0.967	0.947	0.991	0.970	0.977	0.988	0.946	0.974	0.974
**Lactic acid (L)**																
L_m	16.5±1.2^{A}	16.3±1.3^{A}	15.9±1.0^{A}	16.2±0.9^{A}	16.4±0.9^{A}	16.5±0.9^{A}	16.5±0.7^{A}	16.3±0.7^{A}	16.4±0.7^{A}	15.2±1.0^{A}	14.8±0.6^{A}	15.9±0.7^{A}	15.7±0.8^{A}	16.3±1.3^{A}	15.9±1.0^{A}	15.8±1.1^{A}
v_L	1.58±0.43^{A}	1.36±0.35^{A}	1.45±0.33^{A}	1.45±0.27^{A}	1.58±0.32^{A}	1.67±0.36^{A}	1.94±0.39^{A}	1.77±0.35^{A}	1.37±0.19^{A}	1.46±0.33^{A}	1.52±0.24^{A}	1.50±0.25^{A}	1.49±0.29^{A}	1.36±0.35^{A}	1.45±0.33^{A}	1.31±0.33^{A}
λ_L	6.69±1.49^{A}	6.90±1.59^{A}	7.11±1.30^{A}	7.24±1.08^{A}	7.23±1.09^{A}	7.64±1.12^{A}	7.77±0.89^{B}	6.73±0.96^{A}	6.37±0.88^{A}	7.96±1.27^{A}	7.59±0.81^{A}	7.38±0.90^{A}	7.38±1.08^{A}	6.90±1.59^{A}	7.11±1.30^{A}	5.25±1.59^{A}
μ_L	0.38±0.12^{A}	0.33±0.10^{A}	0.37±0.09^{A}	0.36±0.07^{A}	0.39±0.09^{A}	0.41±0.10^{A}	0.47±0.10^{A}	0.43±0.10^{A}	0.34±0.05^{A}	0.38±0.10^{A}	0.41±0.07^{A}	0.38±0.07^{A}	0.38±1.08^{A}	0.33±0.10^{A}	0.37±0.09^{A}	0.33±0.09^{A}
τ_L	11.9±0.9^{A}	12.9±1.1^{A}	12.6±0.9^{A}	12.8±0.7^{A}	12.4±0.7^{A}	12.6±0.7^{A}	12.0±0.5^{A}	11.3±0.6^{A}	12.3±0.6^{A}	13.2±0.9^{B}	12.5±0.5^{A}	12.7±0.6^{A}	12.7±0.7^{A}	12.9±1.1^{A}	12.6±0.9^{A}	11.3±1.0^{A}
t_{mL}	17.1±2.1^{A}	18.9±2.4^{A}	18.1±1.9^{A}	18.4±1.6^{A}	17.6±1.6^{A}	17.5±1.6^{A}	16.3±1.2^{A}	15.9±1.3^{A}	18.3±1.3^{A}	18.4±1.8^{A}	17.4±1.1^{A}	17.9±1.3^{A}	18.0±1.6^{A}	18.9±2.4^{A}	18.1±1.9^{A}	17.3±2.3^{A}
R^2	0.994	0.994	0.996	0.997	0.996	0.996	0.997	0.997	0.998	0.996	0.998	0.998	0.997	0.994	0.996	0.993
**Acetic acid (A)**																
A_m	2.47 (NS)	3.48 (NS)	31.4 (NS)	2.30 (NS)	1.59±0.79^{A}	1.24±0.24^{A}	1.35±1.30^{A}	3.00 (NS)	1.74±1.08^{A}	2.28±1.49^{A}	1.35±0.35^{A}	2.14 (NS)	1.24±0.18^{A}	1.42±0.56^{A}	1.33±0.25^{A}	1.25±0.26^{A}
v_A	0.10 (NS)	0.11 (NS)	0.90 (NS)	0.06±0.02^{A}	0.08±0.02^{A}	0.08±0.02^{A}	0.06±0.05^{A}	0.11 (NS)	0.08±0.04^{A}	0.09±0.03^{A}	0.08±0.05^{A}	0.10±0.07^{A}	0.13±0.05^{B}	0.10±0.02^{B}	0.07±0.01^{A}	0.06±0.01^{A}
λ_A	18.1 (NS)	18.2±12.7^{A}	39.3 (NS)	1.00 (NS)	15.5±2.6^{B}	15.0±1.5^{B}	8.03±7.96^{A}	18.2 (NS)	16.3±3.3^{B}	12.5±4.4^{A}	14.9±1.9^{B}	17.7±7.3^{B}	15.9±1.9^{B}	19.7±1.7^{B}	12.8±1.4^{B}	7.07±2.28^{A}
μ_A	0.13 (NS)	0.13±0.05^{A}	0.11 (NS)	0.10±0.08^{A}	0.21±0.10^{A}	0.26±0.09^{A}	0.19 (NS)	0.69±0.62^{A}	0.19±0.09^{A}	0.16±0.08^{A}	0.25±0.10^{A}	0.19±0.13^{A}	0.43±0.20^{A}	0.24±0.07^{A}	0.21±0.05^{A}	0.18±0.06^{A}
τ_A	39.7 (NS)	33.6±18.6^{A}	57.6 (NS)	21.8 (NS)	25.3±5.9^{A}	22.7±2.1^{A}	18.7±11.2^{A}	21.0±1.6^{A}	26.7±7.3^{A}	25.3±9.4^{A}	22.9±2.9^{A}	28.5±14.1^{A}	20.6±1.3^{A}	27.9±3.6^{B}	22.2±2.3^{A}	18.2±3.3^{A}
t_{mA}	54.7 (NS)	49.0±24.7^{A}	77.8 (NS)	42.6±39.6^{A}	35.0±10.2^{A}	30.4±4.4^{A}	29.3±22.4^{A}	26.2 (NS)	37.1±11.8^{A}	38.2±15.4^{A}	30.9±5.9^{A}	39.2±21.4^{A}	25.3±3.0^{A}	36.1±5.8^{A}	31.6±4.5^{A}	29.3±6.5^{A}
R^2	0.954	0.991	0.953	0.945	0.987	0.994	0.978	0.974	0.990	0.982	0.989	0.978	0.987	0.998	0.995	0.989

Table S3. Numerical values and confidence intervals for parameters obtained from experimental data of Ln modelled by equations (1-4). R^2 is the determination coefficient among experimental and predicted data. NS: not significant. MRS1 and MRS2 are used as control commercial media. Different letters in each row (as superscript) means significant difference between fish peptone media and control media ($p < 0.05$).

	Sk_BW	Sk_RS	Sk_Ma	Sk_Po	Sk_Gu	Sk_Gr	Sk_Me	Sk_Ha	Sk_Bo	Sk_AHM	He_BW	He_RS	He_Ma	He_Po	He_Gu	MRS1
**Biomass (X)**																
X_m	4.22±0.30^{A}	4.69±0.42^{A}	3.88±0.21^{B}	4.80±0.35^{A}	4.75±0.20^{B}	4.61±0.45^{A}	4.82±0.54^{A}	5.03±0.31^{B}	4.32±0.41^{A}	4.50±0.40^{A}	4.11±0.45^{A}	4.22±0.54^{A}	4.28±0.38^{A}	4.49±0.41^{A}	4.41±0.40^{A}	4.32±0.20^{A}
v_m	0.37±0.10^{A}	0.38±0.13^{A}	0.27±0.05^{B}	0.40±0.11^{A}	0.41±0.08^{A}	0.38±0.14^{A}	0.36±0.14^{A}	0.41±0.12^{A}	0.36±0.13^{A}	0.37±0.13^{A}	0.29±0.14^{A}	0.35±0.14^{A}	0.29±0.12^{A}	0.34±0.13^{A}	0.27±0.13^{A}	0.42±0.09^{A}
λ_X	4.90±1.73^{A}	4.51±2.21^{A}	4.58±1.53^{A}	4.53±1.84^{A}	4.68±1.40^{A}	4.24±2.46^{A}	3.81±2.76^{A}	4.57±1.87^{A}	4.58±2.36^{A}	4.54±2.21^{A}	3.65±2.46^{A}	3.88±2.76^{A}	4.53±1.87^{A}	3.84±2.36^{A}	4.51±2.21^{A}	5.55±1.29^{A}
μ_X	0.35±0.11^{A}	0.33±0.12^{A}	0.28±0.07^{A}	0.34±0.10^{A}	0.34±0.11^{A}	0.33±0.13^{A}	0.30±0.13^{A}	0.33±0.10^{A}	0.33±0.11^{A}	0.33±0.12^{A}	0.28±0.13^{A}	0.33±0.13^{A}	0.27±0.10^{A}	0.30±0.13^{A}	0.24±0.12^{A}	0.38±0.10^{A}
τ_X	10.6±1.0^{A}	10.7±1.3^{A}	11.8±1.0^{A}	10.5±1.0^{A}	10.5±1.1^{A}	10.3±1.4^{A}	10.5±1.6^{A}	10.6±1.1^{A}	10.6±1.3^{A}	10.6±1.2^{A}	10.7±1.4^{A}	9.89±1.60^{A}	11.9±1.1^{A}	10.5±1.3^{A}	12.7±1.2^{B}	10.8±0.7^{A}
t_{mX}	16.4±2.2^{A}	16.8±2.9^{A}	19.0±2.4^{A}	16.5±2.4^{A}	16.4±2.4^{A}	16.4±3.2^{A}	17.1±3.8^{A}	16.7±2.5^{A}	16.7±3.1^{A}	16.6±2.9^{A}	17.8±3.2^{A}	15.9±3.8^{A}	19.2±2.5^{A}	17.2±3.1^{A}	20.9±2.9^{B}	16.0±1.6^{A}
R^2	0.993	0.989	0.995	0.992	0.992	0.986	0.983	0.992	0.992	0.988	0.977	0.977	0.978	0.968	0.983	0.995
**Cells (G)**																
G_m	12.9±1.1^{A}	13.1±1.1^{A}	12.4±0.8^{A}	13.3±1.1^{A}	12.6±0.8^{A}	12.8±0.9^{A}	12.7±1.0^{A}	12.1±0.7^{A}	12.7±0.9^{A}	12.8±0.8^{A}	12.3±1.0^{A}	12.5±1.0^{A}	12.3±0.7^{A}	12.5±0.9^{A}	12.1±0.7^{A}	12.7±0.8^{A}
v_G	1.27±0.47^{A}	1.23±0.42^{A}	1.42±0.43^{A}	1.30±0.45^{A}	1.56±0.47^{A}	1.46±0.49^{A}	1.20±0.40^{A}	1.32±0.34^{A}	1.30±0.39^{A}	1.31±0.36^{A}	1.37±0.49^{A}	1.23±0.43^{A}	1.27±0.32^{A}	1.32±0.44^{A}	1.27±0.32^{A}	1.27±0.36^{A}
λ_G	4.00±2.20^{A}	4.01±2.11^{A}	3.99±1.60^{A}	3.78±2.11^{A}	4.76±1.51^{A}	4.59±1.78^{A}	3.84±2.04^{A}	3.88±1.41^{A}	3.90±1.73^{A}	4.50±1.58^{A}	4.11±1.95^{A}	4.09±2.07^{A}	3.84±1.46^{A}	3.80±1.86^{A}	4.11±1.45^{A}	3.71±1.68^{A}
μ_G	0.39±0.16^{A}	0.38±0.14^{A}	0.46±0.15^{A}	0.39±0.15^{A}	0.49±0.16^{A}	0.46±0.16^{A}	0.38±0.14^{A}	0.44±0.12^{A}	0.41±0.13^{A}	0.41±0.12^{A}	0.45±0.17^{A}	0.39±0.15^{A}	0.41±0.11^{A}	0.42±0.15^{A}	0.42±0.11^{A}	0.40±0.12^{A}
τ_G	9.08±1.19^{A}	9.29±1.14^{A}	8.35±0.91^{A}	8.91±1.16^{A}	8.81±0.81^{A}	8.96±0.95^{A}	9.14±1.11^{A}	8.45±0.80^{A}	8.78±0.95^{A}	9.38±0.83^{A}	8.60±1.08^{A}	9.18±1.11^{A}	8.69±0.81^{A}	8.52±1.04^{A}	8.88±0.79^{A}	8.71±0.93^{A}
t_{mG}	14.2±2.5^{A}	14.6±2.4^{A}	12.7±1.7^{A}	14.0±2.4^{A}	12.9±1.5^{A}	13.3±1.8^{A}	14.4±2.3^{A}	13.0±1.5^{A}	13.7±1.9^{A}	14.3±1.7^{A}	13.1±2.1^{A}	14.3±2.3^{A}	13.5±1.6^{A}	13.2±2.0^{A}	13.6±1.6^{A}	13.7±1.9^{A}
R^2	0.986	0.988	0.991	0.987	0.992	0.990	0.988	0.993	0.991	0.993	0.987	0.987	0.993	0.989	0.994	0.991
**Lactic acid (L)**																
L_m	14.9±0.9^{A}	15.5±1.2^{B}	16.5±1.4^{B}	16.9±1.2^{B}	16.4±1.2^{B}	17.1±1.0^{B}	17.1±0.9^{B}	17.7±1.2^{B}	17.8±1.5^{B}	17.5±0.7^{B}	17.6±1.1^{B}	17.4±1.1^{B}	17.3±1.1^{B}	16.5±1.5^{B}	16.8±1.1^{B}	17.1±1.2^{B}
v_L	1.43±0.42^{A}	1.37±0.43^{A}	1.40±0.46^{A}	1.38±0.37^{A}	1.38±0.39^{A}	1.59±0.38^{A}	1.59±0.35^{A}	1.51±0.41^{A}	1.33±0.38^{A}	1.77±0.33^{A}	1.47±0.36^{A}	1.53±0.40^{A}	1.55±0.40^{A}	1.33±0.45^{A}	1.54±0.44^{A}	1.40±0.37^{A}
λ_L	4.03±1.77^{A}	4.51±1.99^{A}	4.07±2.20^{A}	4.45±1.79^{A}	4.41±1.85^{A}	4.38±1.49^{A}	4.67±1.35^{A}	4.17±1.77^{A}	3.98±2.03^{A}	5.11±1.04^{A}	4.39±1.62^{A}	4.26±1.62^{A}	4.10±1.64^{A}	3.82±2.35^{A}	4.19±1.77^{A}	5.46±1.68^{A}
μ_L	0.38±0.12^{A}	0.35±0.12^{A}	0.34±0.12^{A}	0.33±0.10^{A}	0.34±0.11^{A}	0.37±0.10^{A}	0.37±0.09^{A}	0.34±0.10^{A}	0.30±0.10^{A}	0.41±0.08^{A}	0.33±0.09^{A}	0.35±0.10^{A}	0.36±0.10^{A}	0.32±0.12^{A}	0.37±0.11^{A}	0.33±0.10^{A}
τ_L	9.25±0.95^{A}	10.2±1.1^{B}	10.0±1.2^{B}	10.6±1.0^{B}	10.3±1.0^{B}	9.79±0.79^{A}	10.0±0.7^{B}	10.0±1.0^{B}	10.7±1.2^{B}	10.1±0.5^{B}	10.4±1.0^{B}	9.94±0.88^{B}	9.68±0.89^{A}	10.1±1.3^{B}	9.63±0.95^{A}	11.6±1.0^{B}
t_{mL}	14.5±2.0^{A}	15.8±2.4^{A}	15.9±2.7^{A}	16.7±2.3^{A}	16.3±2.4^{A}	15.2±1.8^{A}	15.4±1.6^{A}	15.9±2.2^{A}	17.4±2.8^{A}	15.0±1.2^{A}	16.4±2.1^{A}	15.6±2.0^{A}	15.3±2.0^{A}	16.3±3.0^{A}	15.1±2.1^{A}	17.6±2.4^{A}
R^2	0.992	0.990	0.989	0.993	0.992	0.994	0.996	0.992	0.991	0.997	0.994	0.994	0.993	0.987	0.992	0.994
**Acetic acid (A)**																
A_m	2.04±0.13^{A}	2.03±0.15^{A}	2.34±0.18^{A}	2.16±0.19^{A}	2.13±0.13^{A}	1.82±0.16^{B}	1.83±0.18^{B}	2.08±0.23^{A}	2.16±0.21^{A}	1.97±0.28^{B}	2.18±0.29^{A}	2.34±0.18^{A}	2.38±0.31^{A}	1.57±0.18^{B}	2.26±0.28^{A}	1.56±0.24^{B}
v_A	0.15±0.03^{A}	0.15±0.03^{A}	0.17±0.03^{B}	0.17±0.04^{A}	0.16±0.03^{A}	0.44±0.27^{B}	0.17±0.06^{A}	0.16±0.06^{A}	0.19±0.06^{A}	0.14±0.05^{A}	0.15±0.05^{A}	0.15±0.04^{A}	0.16±0.05^{A}	0.13±0.05^{A}	0.15±0.04^{A}	0.10±0.03^{A}
λ_A	6.11±1.31^{A}	7.40±1.31^{A}	6.44±1.44^{A}	7.54±1.61^{A}	6.56±1.13^{A}	8.87±1.41^{A}	7.30±2.04^{A}	6.16±2.33^{A}	7.78±1.85^{A}	6.40±2.64^{A}	6.57±2.45^{A}	6.92±2.02^{A}	6.51±2.42^{A}	6.90±2.30^{A}	6.49±2.14^{A}	7.73±2.21^{A}
μ_A	0.30±0.07^{A}	0.30±0.07^{A}	0.29±0.07^{A}	0.31±0.09^{A}	0.29±0.06^{A}	0.97±0.61^{B}	0.37±0.15^{A}	0.31±0.13^{A}	0.35±0.13^{A}	0.28±0.12^{A}	0.28±0.11^{A}	0.26±0.08^{A}	0.27±0.11^{A}	0.32±0.14^{A}	0.26±0.09^{A}	0.24±0.09^{A}
τ_A	12.8±0.9^{A}	14.2±1.0^{B}	13.4±1.1^{B}	14.0±1.2^{B}	13.4±0.8^{B}	10.9±0.7^{A}	12.7±1.3^{B}	12.6±1.5^{B}	13.5±1.3^{B}	13.6±2.0^{B}	13.7±1.9^{B}	14.6±1.7^{B}	13.8±1.9^{B}	13.1±1.6^{B}	14.2±1.7^{B}	16.0±2.1^{B}
t_{mA}	19.5±2.1^{A}	20.9±2.2^{A}	20.4±2.4^{A}	20.4±2.7^{A}	20.2±1.9^{A}	13.0±1.5^{B}	18.1±3.0^{A}	19.0±3.6^{A}	19.2±2.9^{A}	20.7±4.5^{A}	20.9±4.2^{A}	22.4±3.8^{A}	21.1±4.2^{A}	19.3±3.6^{A}	21.9±3.9^{A}	24.2±4.6^{A}
R^2	0.996	0.996	0.995	0.993	0.997	0.985	0.988	0.987	0.990	0.983	0.986	0.991	0.987	0.986	0.989	0.989

Table S4. Continuation of Table S3.

	He_Gr	He_Bo	He_Ha	He_AHM	He_Me	Wh_BW	Wh_RS	Wh_Ma	Wh_Po	Wh_Gu	Wh_Gr	Wh_Bo	Wh_Ha	Wh_Me	Wh_AHM	MRS2
**Biomass (X)**																
X_m	4.30±0.42^{A}	4.74±0.53^{A}	4.70±0.64^{A}	4.24±0.44^{A}	4.12±0.39^{A}	4.64±0.45^{A}	4.67±0.31^{A}	4.57±0.53^{A}	4.82±0.44^{A}	4.60±0.39^{A}	4.73±0.38^{A}	4.73±0.44^{A}	4.86±0.45^{A}	4.79±0.51^{A}	4.66±0.32^{A}	4.47±0.21^{A}
v_m	0.34±0.12^{A}	0.36±0.14^{A}	0.30±0.11^{A}	0.33±0.12^{A}	0.35±0.13^{A}	0.33±0.10^{A}	0.38±0.11^{A}	0.33±0.12^{A}	0.36±0.11^{A}	0.36±0.10^{A}	0.37±0.11^{A}	0.38±0.13^{A}	0.37±0.12^{A}	0.35±0.12^{A}	0.38±0.10^{A}	0.40±0.11^{A}
λ_X	3.78±2.47^{A}	3.81±2.77^{A}	3.71±3.02^{A}	4.09±2.56^{A}	3.83±2.45^{A}	4.03±2.26^{A}	4.49±1.97^{A}	3.98±2.78^{A}	4.39±0.22^{A}	4.48±2.00^{A}	3.91±2.01^{A}	3.70±2.35^{A}	3.88±2.31^{A}	3.86±2.58^{A}	3.79±1.77^{A}	4.86±1.65^{A}
μ_X	0.31±0.12^{A}	0.31±0.13^{A}	0.26±0.11^{A}	0.31±0.13^{A}	0.34±0.14^{A}	0.28±0.10^{A}	0.33±0.11^{A}	0.29±0.12^{A}	0.30±0.11^{A}	0.31±0.10^{A}	0.31±0.10^{A}	0.32±0.12^{A}	0.30±0.11^{A}	0.29±0.12^{A}	0.32±0.10^{A}	0.36±0.11^{A}
τ_X	10.2±1.4^{A}	10.3±1.6^{A}	11.5±2.1^{A}	10.6±1.5^{A}	9.79±1.37^{A}	11.2±1.4^{A}	10.6±1.1^{A}	10.9±1.7^{A}	11.0±1.3^{A}	11.0±1.2^{A}	11.0±1.2^{A}	9.97±1.34^{A}	10.5±1.4^{A}	10.7±1.6^{A}	9.98±1.20^{A}	10.4±0.9^{A}
t_{mX}	16.5±3.2^{A}	16.9±7.0^{A}	19.3±4.8^{A}	17.1±3.5^{A}	15.8±3.1^{A}	18.3±3.3^{A}	16.7±4.1^{A}	17.9±4.0^{A}	17.6±3.1^{A}	17.4±2.8^{A}	16.7±2.7^{A}	16.2±3.0^{A}	17.1±3.1^{A}	17.6±3.6^{A}	16.2±2.3^{A}	15.9±2.0^{A}
R^2	0.985	0.982	0.981	0.984	0.984	0.989	0.991	0.983	0.989	0.991	0.991	0.987	0.988	0.985	0.993	0.993
**Cells (G)**																
G_m	13.2±1.1^{A}	13.1±1.0^{A}	12.8±0.9^{A}	12.6±0.9^{A}	13.1±1.5^{A}	12.9±1.1^{A}	12.7±1.0^{A}	12.4±0.9^{A}	12.3±1.0^{A}	12.7±1.6^{A}	12.5±1.1^{A}	12.6±0.8^{A}	12.2±0.9^{A}	12.3±0.9^{A}	12.2±1.5^{A}	12.8±0.9^{A}
v_G	1.26±0.43^{A}	1.24±0.39^{A}	1.15±0.32^{A}	1.22±0.32^{A}	0.93±0.34^{A}	1.15±0.40^{A}	1.13±0.37^{A}	1.12±0.35^{A}	1.22±0.45^{A}	0.95±0.41^{A}	1.10±0.38^{A}	1.25±0.34^{A}	1.09±0.33^{A}	1.35±0.43^{A}	0.97±0.44^{A}	1.18±0.34^{A}
λ_G	4.13±2.04^{A}	4.13±1.90^{A}	3.76±1.81^{A}	3.96±1.81^{A}	4.08±2.71^{A}	3.90±2.24^{A}	3.92±2.08^{A}	4.04±1.94^{A}	3.64±2.19^{A}	3.30±3.18^{A}	3.73±2.24^{A}	3.98±1.63^{A}	3.30±1.98^{A}	3.95±1.75^{A}	3.44±3.21^{A}	3.80±1.79^{A}
μ_G	0.38±0.14^{A}	0.38±0.13^{A}	0.36±0.11^{A}	0.39±0.13^{A}	0.28±0.11^{A}	0.36±0.14^{A}	0.36±0.13^{A}	0.36±0.11^{A}	0.40±0.13^{A}	0.30±0.12^{A}	0.35±0.13^{A}	0.40±0.12^{A}	0.36±0.12^{A}	0.44±0.15^{A}	0.32±0.16^{A}	0.37±0.11^{A}
τ_G	9.37±1.11^{A}	9.40±1.09^{A}	9.35±0.99^{A}	9.10±0.98^{A}	11.1±1.7^{A}	9.50±1.12^{A}	9.53±1.02^{A}	9.56±0.99^{A}	8.66±0.98^{A}	10.0±1.7^{A}	9.44±1.24^{A}	9.00±0.88^{A}	8.92±1.11^{A}	8.50±0.98^{A}	9.75±1.83^{A}	9.21±0.98^{A}
t_{mG}	14.6±2.4^{A}	14.7±2.2^{A}	14.9±2.1^{A}	14.2±2.0^{A}	18.1±4.0^{A}	14.8±2.4^{A}	15.1±2.2^{A}	15.1±2.1^{A}	13.7±2.0^{A}	16.7±4.0^{A}	15.1±2.7^{A}	14.0±1.8^{A}	14.5±2.6^{A}	13.0±1.9^{A}	16.1±4.1^{A}	14.6±2.1^{A}
R^2	0.987	0.990	0.991	0.991	0.982	0.986	0.989	0.990	0.985	0.976	0.986	0.992	0.989	0.990	0.974	0.991
**Lactic acid (L)**																
L_m	16.2±0.9^{A}	17.3±1.0^{A}	17.5±1.0^{A}	17.7±2.1^{A}	17.5±1.5^{A}	17.5±1.1^{A}	17.2±1.3^{A}	16.5±1.4^{A}	16.8±1.1^{A}	17.3±0.6^{A}	17.0±0.9^{A}	17.2±1.0^{A}	17.4±1.1^{A}	17.3±1.2^{A}	17.2±1.2^{A}	16.9±1.8^{A}
v_L	1.48±0.36^{A}	1.51±0.37^{A}	1.57±0.37^{A}	1.21±0.42^{A}	1.42±0.44^{A}	1.48±0.37^{A}	1.53±0.55^{A}	1.59±0.50^{A}	1.49±0.38^{A}	1.64±0.25^{A}	1.49±0.32^{A}	1.54±0.36^{A}	1.57±0.40^{A}	1.49±0.40^{A}	1.54±0.42^{A}	1.48±0.45^{A}
λ_L	4.87±1.47^{A}	4.48±1.54^{A}	4.43±1.47^{A}	3.75±2.72^{A}	4.32±2.10^{A}	4.65±1.61^{A}	4.61±2.05^{A}	4.52±2.04^{A}	4.73±1.58^{A}	5.40±0.89^{A}	4.63±1.36^{A}	4.35±1.49^{A}	4.48±1.59^{A}	4.41±1.78^{A}	4.72±1.70^{A}	4.71±1.60^{A}
μ_L	0.37±0.10^{A}	0.35±0.09^{A}	0.36±0.09^{A}	0.27±0.11^{A}	0.32±0.11^{A}	0.34±0.09^{A}	0.36±0.10^{A}	0.39±0.12^{A}	0.36±0.10^{A}	0.38±0.06^{A}	0.35±0.08^{A}	0.36±0.09^{A}	0.36±0.10^{A}	0.34±0.10^{A}	0.36±0.11^{A}	0.37±0.09^{A}
τ_L	10.3±0.8^{A}	10.2±0.8^{A}	10.0±0.8^{A}	11.1±1.7^{A}	10.5±1.2^{A}	10.6±0.9^{A}	10.2±0.9^{A}	9.70±0.88^{A}	10.4±0.9^{A}	10.7±0.5^{A}	10.3±0.7^{A}	9.94±0.30^{A}	10.0±0.9^{A}	10.2±1.0^{A}	10.3±0.9^{A}	9.99±0.99^{A}
t_{mL}	15.8±2.1^{A}	15.9±2.0^{A}	15.6±2.0^{A}	18.4±3.0^{A}	16.7±2.1^{A}	16.5±2.1^{A}	15.8±2.0^{A}	14.9±1.9^{A}	16.0±2.0^{A}	15.9±1.1^{A}	16.0±1.7^{A}	15.5±1.8^{A}	15.6±1.9^{A}	16.0±2.2^{A}	15.9±2.1^{A}	15.9±2.1^{A}
R^2	0.995	0.994	0.995	0.984	0.990	0.994	0.993	0.993	0.994	0.998	0.996	0.995	0.994	0.993	0.993	0.994
**Acetic acid (A)**																
A_m	1.43±0.15^{A}	1.96±0.20^{B}	2.14±0.20^{B}	1.25±0.12^{B}	2.00±0.18^{B}	2.32±0.45^{B}	2.29±0.22^{B}	2.39±0.27^{B}	2.12±0.20^{B}	2.11±0.21^{B}	1.98±0.15^{B}	2.00±0.17^{B}	2.46±0.27^{B}	2.16±0.19^{B}	2.35±0.27^{B}	1.63±0.12^{A}
v_A	0.13±0.04^{A}	0.12±0.03^{A}	0.15±0.04^{A}	0.18±0.12^{A}	0.14±0.04^{A}	0.14±0.05^{A}	0.16±0.04^{A}	0.17±0.05^{A}	0.15±0.04^{A}	0.15±0.04^{A}	0.18±0.05^{A}	0.19±0.06^{A}	0.18±0.05^{A}	0.16±0.04^{A}	0.17±0.05^{A}	0.11±0.06^{A}
λ_A	9.89±1.76^{A}	5.98±1.77^{A}	6.32±1.80^{A}	7.76±2.29^{A}	6.27±1.76^{A}	6.55±3.07^{A}	7.14±1.75^{A}	7.15±1.96^{A}	6.93±1.68^{A}	6.16±1.92^{A}	7.63±1.50^{A}	7.39±1.72^{A}	7.02±2.04^{A}	6.81±1.70^{A}	6.84±2.14^{A}	7.78±1.88^{A}
μ_A	0.36±0.12^{A}	0.25±0.07^{A}	0.29±0.09^{A}	0.58±0.39^{A}	0.29±0.08^{A}	0.24±0.12^{A}	0.29±0.09^{A}	0.28±0.09^{A}	0.28±0.08^{A}	0.28±0.09^{A}	0.36±0.11^{A}	0.38±0.14^{A}	0.30±0.11^{A}	0.30±0.09^{A}	0.29±0.11^{A}	0.23±0.12^{A}
τ_A	15.5±1.3^{A}	14.0±1.5^{A}	13.2±1.3^{A}	11.2±1.0^{B}	13.3±1.3^{A}	14.9±2.8^{A}	14.1±1.4^{A}	14.3±1.6^{A}	14.0±1.3^{A}	13.4±1.4^{A}	13.2±1.0^{B}	12.7±1.1^{B}	13.8±1.5^{A}	13.4±1.2^{A}	13.7±1.6^{A}	16.3±1.8^{A}
t_{mA}	21.1±2.8^{A}	22.0±3.3^{A}	20.2±3.0^{A}	14.7±2.7^{B}	20.3±2.9^{A}	23.2±6.2^{A}	21.1±3.0^{A}	21.5±3.5^{A}	21.1±2.9^{A}	20.6±3.3^{A}	18.8±2.3^{B}	17.9±2.5^{B}	20.5±3.4^{A}	20.0±2.8^{A}	20.6±3.6^{A}	24.2±2.9^{A}
R^2	0.991	0.993	0.993	0.982	0.993	0.980	0.993	0.991	0.994	0.992	0.994	0.991	0.990	0.993	0.989	0.979

Table S5. Numerical values and confidence intervals for parameters obtained from experimental data of Lb 3 modelled by equations (1-4). R^2 is the determination coefficient among experimental and predicted data. NS: not significant. MRS1 and MRS2 are used as control commercial media. Different letters in each row (as superscript) means significant difference between fish peptone media and control media ($p < 0.05$).

	Sk_BW	Sk_RS	Sk_Ma	Sk_Po	Sk_Gu	Sk_Gr	Sk_Me	Sk_Ha	Sk_Bo	Sk_AHM	He_BW	He_RS	He_Ma	He_Po	He_Gu	MRS1
**Biomass (X)**																
X_m	3.33±0.19^{A}	3.19±0.15^{A}	3.10±0.23^{A}	3.55±0.16^{A}	2.87±0.35^{A}	3.07±0.18^{A}	2.88±0.07^{B}	3.63±0.27^{A}	3.73±0.28^{B}	3.44±0.41^{A}	3.72±0.27^{B}	3.66±0.40^{A}	3.30±0.23^{A}	3.69±0.10^{B}	3.48±0.08^{A}	3.19±0.22^{A}
v_m	0.37±0.09^{A}	0.43±0.11^{A}	0.31±0.08^{A}	0.39±0.08^{A}	0.25±0.10^{A}	0.33±0.08^{A}	0.30±0.03^{A}	0.48±0.17^{A}	0.42±0.12^{A}	0.30±0.11^{A}	0.56±0.24^{A}	0.40±0.19^{A}	0.36±0.14^{A}	0.44±0.06^{A}	0.42±0.05^{A}	0.37±0.12^{A}
λ_X	8.53±1.11^{A}	8.15±0.99^{A}	9.53±1.39^{A}	7.39±0.92^{A}	7.66±2.43^{A}	7.25±1.23^{A}	8.24±0.49^{A}	9.17±1.43^{A}	9.37±1.42^{A}	8.54±2.28^{A}	8.97±1.41^{A}	8.12±2.18^{A}	8.00±2.32^{A}	7.97±0.57^{A}	8.20±0.47^{A}	9.68±1.53^{A}
μ_X	0.45±0.11^{A}	0.54±0.15^{A}	0.40±0.11^{A}	0.44±0.09^{A}	0.35±0.16^{A}	0.43±0.11^{A}	0.41±0.04^{A}	0.53±0.20^{A}	0.45±0.14^{A}	0.35±0.15^{A}	0.60±0.27^{A}	0.44±0.22^{A}	0.44±0.17^{A}	0.47±0.07^{A}	0.48±0.06^{A}	0.47±0.17^{A}
τ_X	13.0±0.7^{A}	11.9±0.6^{B}	14.6±0.9^{A}	11.9±0.6^{B}	13.4±1.7^{A}	11.9±0.8^{B}	13.1±0.3^{A}	13.0±0.9^{A}	13.8±0.9^{A}	14.2±1.6^{A}	12.3±0.8^{A}	12.7±1.4^{A}	12.6±1.9^{A}	12.2±0.4^{B}	12.4±0.3^{B}	14.0±1.0^{A}
t_{mX}	17.5±1.6^{A}	15.6±1.3^{A}	19.6±2.0^{A}	16.5±1.3^{A}	19.2±3.7^{A}	16.6±1.7^{A}	17.9±0.7^{A}	16.8±2.0^{A}	18.2±1.9^{A}	19.9±3.4^{A}	15.6±1.9^{A}	17.2±3.1^{A}	16.4±2.5^{A}	16.4±0.8^{A}	16.6±0.7^{A}	18.2±2.0^{A}
R^2	0.996	0.996	0.995	0.997	0.984	0.995	0.999	0.993	0.994	0.987	0.992	0.985	0.990	0.999	0.999	0.992
**Cells (G)**																
G_m	13.3±1.7^{A}	12.5±1.7^{A}	13.5±0.7^{A}	12.9±1.4^{A}	12.7±1.4^{A}	12.6±1.7^{A}	12.8±1.9^{A}	12.1±1.8^{A}	12.6±1.4^{A}	11.9±1.6^{A}	12.3±1.9^{A}	12.1±1.1^{A}	12.5±1.6^{A}	13.3±1.5^{A}	12.9±1.8^{A}	12.1±1.3^{A}
v_G	1.51±0.78^{A}	1.51±0.94^{A}	1.34±0.54^{A}	1.79±0.81^{A}	1.41±0.90^{A}	1.54±1.09^{A}	1.27±0.78^{A}	1.34±0.94^{A}	1.43±0.81^{A}	1.44±0.90^{A}	1.53±1.09^{A}	1.91±1.06^{A}	1.84±0.87^{A}	1.69±0.63^{A}	1.48±0.58^{A}	1.31±0.51^{A}
λ_G	4.05±3.41^{A}	4.05±3.50^{A}	2.94(NS)	4.74±2.77^{A}	3.56±2.90^{A}	5.01±3.25^{A}	2.94(NS)	3.56±3.50^{A}	4.05±2.77^{A}	4.08±2.90^{A}	4.24±3.25^{A}	5.33±2.01^{A}	5.07±2.54^{A}	4.94±2.13^{A}	5.10±2.31^{A}	5.18±1.68^{A}
μ_G	0.45±0.26^{A}	0.48±0.33^{A}	0.40±0.18^{A}	0.55±0.27^{A}	0.44±0.32^{A}	0.49±0.37^{A}	0.40±0.26^{A}	0.44±0.33^{A}	0.45±0.27^{A}	0.48±0.32^{A}	0.49±0.37^{A}	0.63±0.36^{A}	0.59±0.36^{A}	0.51±0.17^{A}	0.46±0.15^{A}	0.44±0.13^{A}
τ_G	8.45±1.90^{A}	8.19±1.93^{A}	7.97±0.69^{B}	8.35±1.52^{A}	8.08±1.58^{A}	9.12±1.77^{A}	7.97±1.90^{A}	8.08±1.93^{A}	8.45±1.52^{A}	8.19±1.58^{A}	8.29±1.77^{A}	8.52±1.04^{A}	8.47±1.04^{A}	8.87±1.24^{A}	9.48±1.44^{A}	9.78±1.02^{A}
t_{mG}	12.9±4.2^{A}	12.3±4.2^{A}	13.0±1.5^{A}	12.0±3.3^{A}	12.6±3.4^{A}	13.3±3.8^{A}	13.0±4.2^{A}	12.6±4.0^{A}	12.9±3.3^{A}	12.3±3.4^{A}	12.3±3.8^{A}	11.7±2.2^{A}	11.9±2.2^{A}	12.8±2.8^{A}	13.9±3.3^{A}	13.9±2.3^{A}
R^2	0.972	0.968	0.962	0.993	0.956	0.982	0.962	0.956	0.972	0.968	0.959	0.981	0.978	0.995	0.996	0.989
**Lactic acid (L)**																
L_m	17.3±1.1^{A}	16.6±1.6^{A}	16.5±1.4^{A}	17.9±1.9^{A}	16.3±1.8^{A}	17.1±1.9^{A}	17.0±1.0^{A}	18.7±1.4^{A}	18.1±1.1^{A}	17.3±0.9^{A}	17.2±1.4^{A}	17.4±1.7^{A}	16.6±1.0^{A}	17.9±1.3^{A}	18.0±1.5^{A}	17.1±2.1^{A}
v_L	1.57±0.28^{A}	1.68±0.30^{A}	1.52±0.36^{A}	1.45±0.39^{A}	1.45±0.67^{A}	1.47±0.62^{A}	1.51±0.29^{A}	1.50±0.33^{A}	1.63±0.34^{A}	1.75±0.34^{A}	1.58±0.47^{A}	1.53±0.52^{A}	1.84±0.46^{A}	1.96±0.54^{A}	2.16±0.80^{A}	1.98±0.61^{A}
λ_L	7.62±1.12^{A}	7.61±1.19^{A}	7.42±1.20^{A}	7.69±1.42^{A}	7.71±1.78^{A}	7.53±2.32^{A}	7.69±1.13^{A}	7.71±1.42^{A}	7.62±1.22^{A}	7.61±1.02^{A}	7.42±1.70^{A}	6.91±2.02^{A}	9.22±1.21^{A}	9.31±1.36^{A}	8.69±1.62^{A}	8.58±2.16^{A}
μ_L	0.36±0.06^{A}	0.40±0.09^{A}	0.37±0.12^{A}	0.32±0.13^{A}	0.36±0.17^{A}	0.34±0.15^{A}	0.36±0.08^{A}	0.32±0.09^{A}	0.36±0.11^{A}	0.40±0.09^{A}	0.37±0.12^{A}	0.35±0.12^{A}	0.45±0.12^{A}	0.44±0.13^{A}	0.48±0.19^{A}	0.46±0.15^{A}
τ_L	13.2±0.9^{A}	12.6±1.5^{A}	12.9±1.6^{A}	13.9±2.6^{A}	13.3±3.1^{A}	13.4±2.5^{A}	13.3±0.8^{A}	13.9±1.0^{A}	13.2±0.8^{A}	12.6±0.7^{A}	12.9±1.1^{A}	12.6±1.4^{A}	13.7±0.8^{A}	13.9±0.9^{A}	12.9±1.0^{A}	12.9±0.8^{A}
t_{mL}	18.7±1.8^{A}	17.5±2.0^{A}	18.3±1.8^{A}	20.0±1.9^{A}	19.0±2.3^{A}	19.2±3.4^{A}	19.0±1.7^{A}	20.0±2.2^{A}	18.7±1.8^{A}	17.5±1.5^{A}	18.3±2.5^{A}	18.3±3.0^{A}	18.2±1.7^{A}	18.4±1.9^{A}	17.0±2.3^{A}	17.2±1.7^{A}
R^2	0.996	0.997	0.991	0.995	0.997	0.992	0.997	0.995	0.996	0.997	0.991	0.989	0.996	0.995	0.991	0.994
**Acetic acid (A)**																
A_m	0.89±0.15^{A}	1.71(NS)	1.18±0.25^{A}	0.96±0.41^{A}	1.26±0.42^{A}	1.29±0.27^{A}	1.58±1.29^{A}	1.08±0.34^{A}	2.22(NS)	1.08±0.31^{A}	2.12(NS)	4.18(NS)	1.02±0.25^{A}	0.82±0.11^{A}	1.08±0.79^{A}	1.39±0.61^{A}
v_A	0.07±0.01^{A}	0.07±0.03^{A}	0.08±0.01^{A}	0.06±0.05^{A}	0.07±0.03^{A}	0.08±0.02^{A}	0.04±0.01^{A}	0.06±0.03^{A}	0.08±0.07^{A}	0.05±0.01^{A}	0.06±0.04^{A}	0.12(NS)	0.06±0.03^{A}	0.06±0.01^{A}	0.05±0.02^{A}	0.08±0.03^{A}
λ_A	7.98±2.03^{A}	12.0±4.1^{A}	9.25±2.61^{A}	8.22±3.66^{A}	8.34±2.93^{A}	6.62±2.12^{A}	0.10(NS)	9.07±4.31^{A}	17.5±10.6^{A}	7.38±2.86^{A}	13.1±10.4^{A}	22.8(NS)	9.56±3.46^{A}	11.9±1.9^{A}	13.1±4.5^{A}	9.32±2.80^{A}
μ_A	0.30±0.12^{A}	0.17±0.08^{A}	0.26±0.14^{A}	0.23±0.12^{A}	0.23±0.11^{A}	0.24±0.13^{A}	0.11±0.09^{A}	0.24±0.16^{A}	0.15±0.08^{A}	0.18±0.07^{A}	0.12±0.06^{A}	0.12±0.06^{A}	0.25±0.14^{A}	0.28±0.09^{A}	0.17±0.11^{A}	0.24±0.10^{A}
τ_A	14.7±2.6^{A}	24.1±4.1^{A}	16.9±3.2^{A}	16.9±3.9^{A}	17.2±4.6^{A}	15.0±2.9^{A}	19.2±18.1^{A}	17.5±4.3^{A}	30.9±17.3^{A}	18.8±4.5^{A}	29.6±18.1^{A}	39.3±38.3^{A}	17.6±3.3^{A}	19.2±1.7^{A}	24.7±9.9^{A}	17.8±5.0^{A}
t_{mA}	21.4±6.1^{A}	36.2±7.3^{A}	24.6±8.4^{A}	25.5±5.9^{A}	26.1±4.7^{A}	23.4±6.2^{A}	38.3±33.0^{A}	25.9±9.0^{A}	44.4±24.2^{A}	30.3±8.8^{A}	46.1±26.2^{A}	82.7(NS)	25.7±7.1^{A}	26.4±3.7^{A}	36.4±16.9^{A}	26.3±5.4^{A}
R^2	0.988	0.979	0.996	0.915	0.968	0.985	0.939	0.955	0.984	0.981	0.981	0.988	0.975	0.992	0.973	0.982

Table S6. Continuation of Table S5.

	He_Gr	He_Me	He_Ha	He_Bo	He_AHM	Wh_BW	Wh_RS	Wh_Ma	Wh_Po	Wh_Gu	Wh_Gr	Wh_Bo	Wh_Ha	Wh_Me	Wh_AHM	MRS2
**Biomass (X)**																
X_m	3.17±0.27^{A}	3.35±0.14^{A}	2.82±0.43^{A}	3.30±0.20^{A}	3.33±0.22^{A}	3.49±0.31^{A}	3.60±0.29^{A}	3.41±0.30^{A}	3.23±0.19^{A}	3.35±0.19^{A}	3.02±0.27^{A}	3.60±0.28^{B}	3.21±0.41^{A}	3.29±0.27^{A}	3.13±0.40^{A}	3.02±0.29^{A}
v_m	0.39±0.15^{A}	0.38±0.07^{A}	0.22±0.09^{A}	0.40±0.10^{A}	0.33±0.12^{A}	0.36±0.09^{A}	0.47±0.14^{A}	0.38±0.10^{A}	0.43±0.19^{A}	0.75±0.41^{A}	0.30±0.17^{A}	0.33±0.12^{A}	0.30±0.11^{A}	0.31±0.24^{A}	0.29±0.19^{A}	0.31±0.09^{A}
λ_X	8.70±1.68^{A}	8.30±0.85^{A}	8.15±2.79^{A}	9.28±1.50^{A}	9.57±1.32^{A}	8.36±2.08^{A}	7.58±1.21^{A}	8.19±1.68^{A}	8.41±1.91^{A}	9.66±1.28^{A}	9.53±1.43^{A}	7.21±1.42^{A}	8.38±2.28^{A}	7.66±1.41^{A}	8.46±2.18^{A}	9.32±1.09^{A}
μ_X	0.49±0.20^{A}	0.45±0.09^{A}	0.31±0.15^{A}	0.48±0.17^{A}	0.39±0.09^{A}	0.41±0.07^{A}	0.52±0.12^{A}	0.44±0.11^{A}	0.53±0.12^{A}	0.89±0.50^{A}	0.40±0.20^{A}	0.35±0.14^{A}	0.37±0.15^{A}	0.38±0.27^{A}	0.37±0.22^{A}	0.40±0.11^{A}
τ_X	12.8±1.1^{A}	12.7±0.5^{A}	14.6±2.1^{A}	13.4±1.0^{A}	14.7±1.2^{A}	13.2±1.1^{A}	11.4±1.2^{B}	12.7±1.3^{A}	12.2±0.9^{A}	11.9±0.5^{B}	14.6±0.9^{A}	12.9±1.2^{A}	13.8±1.6^{A}	12.9±0.8^{A}	13.9±1.4^{A}	14.3±1.6^{A}
t_{mX}	16.9±2.3^{A}	17.2±1.2^{A}	21.0±4.5^{A}	17.6±1.9^{A}	19.8±2.1^{A}	18.1±2.0^{A}	15.2±1.6^{B}	17.2±1.8^{A}	16.0±1.4^{A}	14.1±1.6^{B}	19.6±2.0^{A}	18.6±1.9^{A}	19.2±3.4^{A}	18.2±1.9^{A}	19.4±3.1^{A}	19.2±2.1^{A}
R^2	0.990	0.998	0.979	0.991	0.998	0.998	0.998	0.985	0.994	0.995	0.998	0.995	0.998	0.995	0.994	0.997
**Cells (G)**																
G_m	13.1±1.7^{A}	12.8±1.1^{A}	12.5±1.0^{A}	12.7±1.9^{A}	13.6±2.0^{A}	13.7±0.7^{A}	12.4±1.4^{A}	12.7±1.9^{A}	12.5±1.8^{A}	13.4±1.6^{A}	12.5±1.9^{A}	12.9±0.7^{A}	12.9±1.4^{A}	13.2±1.4^{A}	12.7±1.9^{A}	12.7±1.1^{A}
v_G	1.60±0.53^{A}	1.93±0.68^{A}	1.52±0.56^{A}	1.51±0.78^{A}	1.69±0.94^{A}	1.88±0.54^{A}	1.45±0.81^{A}	1.54±1.09^{A}	1.39±0.78^{A}	2.14±0.83^{A}	1.80±0.94^{A}	1.54±0.54^{A}	1.26±0.81^{A}	1.23±0.90^{A}	1.54±1.10^{A}	1.53±1.06^{A}
λ_G	5.25±2.30^{A}	6.27±1.38^{A}	5.23±1.40^{A}	5.76±3.41^{A}	5.65±3.50^{A}	6.01±1.29^{A}	5.86±2.77^{A}	6.19±3.25^{A}	6.13±3.41^{A}	5.78±2.17^{A}	6.44±3.50^{A}	6.12±1.29^{A}	5.90±2.77^{A}	5.73±2.90^{A}	6.06±3.25^{A}	5.13±2.01^{A}
μ_G	0.49±0.14^{A}	0.60±0.17^{A}	0.49±0.14^{A}	0.48±0.26^{A}	0.50±0.33^{A}	0.55±0.18^{A}	0.47±0.27^{A}	0.48±0.37^{A}	0.45±0.26^{A}	0.64±0.20^{A}	0.58±0.33^{A}	0.48±0.18^{A}	0.39±0.27^{A}	0.37±0.32^{A}	0.49±0.37^{A}	0.48±0.36^{A}
τ_G	9.34±1.47^{A}	9.59±0.77^{A}	9.33±0.79^{A}	9.98±1.90^{A}	9.65±1.93^{A}	9.66±0.69^{A}	10.2±1.5^{A}	10.3±1.8^{A}	10.6±1.9^{A}	8.92±1.25^{A}	9.91±1.93^{A}	10.3±0.7^{A}	11.0±1.5^{A}	11.1±1.6^{A}	10.2±1.8^{A}	9.29±1.04^{A}
t_{mG}	13.4±3.8^{A}	12.9±1.7^{A}	13.4±1.8^{A}	14.2±4.2^{A}	13.7±4.0^{A}	13.3±1.5^{A}	14.5±3.3^{A}	14.6±3.8^{A}	15.1±4.2^{A}	12.9±2.8^{A}	13.4±3.9^{A}	14.5±1.5^{A}	16.1±3.3^{A}	16.5±3.4^{A}	14.8±3.8^{A}	13.5±2.2^{A}
R^2	0.995	0.999	0.989	0.987	0.992	0.996	0.992	0.994	0.983	0.997	0.988	0.991	0.990	0.988	0.993	0.984
**Lactic acid (L)**																
L_m	17.1±1.2^{A}	17.6±1.3^{A}	18.1±1.5^{A}	17.1±1.0^{A}	17.1±1.8^{A}	17.4±2.5^{A}	17.4±1.9^{A}	18.0±2.3^{A}	17.4±2.3^{A}	16.4±1.2^{A}	16.1±1.5^{A}	17.8±1.4^{A}	18.1±1.9^{A}	17.7±2.2^{A}	16.9±1.6^{A}	17.5±1.1^{A}
v_L	2.67±1.11^{A}	2.05±0.67^{A}	2.33±0.82^{A}	1.74±0.59^{A}	1.60±0.64^{A}	1.67±0.51^{A}	1.71±1.01^{A}	1.81±0.87^{A}	2.12±0.89^{A}	1.38±0.72^{A}	1.57±0.46^{A}	1.68±0.55^{A}	1.69±1.01^{A}	2.10±0.96^{A}	1.88±0.82^{A}	1.82±0.41^{A}
λ_L	8.89±1.36^{A}	8.07±1.47^{A}	8.39±1.41^{A}	9.59±1.91^{A}	10.0±1.66^{A}	9.09±1.82^{A}	8.85±2.36^{A}	8.70±2.47^{A}	8.25±2.41^{A}	8.90±1.61^{A}	9.44±1.52^{A}	8.26±2.06^{A}	10.0±1.8^{A}	8.41±2.17^{A}	8.98±2.32^{A}	9.15±1.16^{A}
μ_L	0.62±0.27^{A}	0.47±0.16^{A}	0.52±0.19^{A}	0.41±0.13^{A}	0.37±0.12^{A}	0.38±0.15^{A}	0.39±0.12^{A}	0.40±0.09^{A}	0.49±0.16^{A}	0.34±0.13^{A}	0.39±0.19^{A}	0.38±0.12^{A}	0.37±0.19^{A}	0.48±0.21^{A}	0.45±0.10^{A}	0.42±0.10^{A}
τ_L	12.1±0.8^{A}	12.4±0.9^{B}	12.3±0.8^{B}	14.5±1.6^{B}	15.4±1.9^{B}	14.3±2.0^{B}	13.9±1.4^{B}	13.7±1.3^{B}	12.3±1.1^{B}	14.9±0.8^{B}	14.6±0.9^{B}	13.6±1.0^{B}	15.4±0.9^{B}	12.6±1.3^{B}	13.5±0.8^{B}	13.9±0.9^{B}
t_{mL}	15.3±1.8^{A}	18.7±2.0^{A}	16.1±1.9^{A}	19.4±2.3^{A}	20.7±2.6^{A}	19.5±2.1^{A}	19.0±1.8^{A}	18.6±2.4^{A}	16.4±1.6^{A}	20.8±2.4^{A}	19.7±2.6^{A}	18.9±1.9^{A}	20.7±2.3^{A}	16.8±1.8^{A}	18.0±2.3^{A}	18.7±1.6^{A}
R^2	0.993	0.993	0.994	0.994	0.995	0.993	0.991	0.996	0.996	0.988	0.992	0.997	0.986	0.998	0.995	0.996
**Acetic acid (A)**																
A_m	1.16±0.85^{A}	1.22±0.65^{A}	0.82±0.21^{A}	1.25±0.34^{A}	1.26±0.42^{A}	1.49±0.57^{A}	1.60±0.71^{A}	1.48±0.81^{A}	1.32±0.77^{A}	1.14±0.58^{A}	1.92(NS)	1.96(NS)	0.71±0.09^{A}	2.00(NS)	1.22±0.36^{A}	0.84±0.53^{A}
v_A	0.05±0.04^{A}	0.05±0.01^{A}	0.06±0.02^{A}	0.08±0.04^{A}	0.09±0.03^{B}	0.09±0.04^{B}	0.15±0.07^{B}	0.08±0.03^{B}	0.08±0.04^{A}	0.07±0.06^{A}	0.09±0.05^{A}	0.08±0.04^{A}	0.07±0.03^{A}	0.08±0.04^{A}	0.07±0.02^{B}	0.03±0.01^{A}
λ_A	14.0±4.9	20.2±0.3	12.0±3.2	9.70±4.01	10.7±3.6	9.02±2.70	9.20±3.20	7.78±2.80	7.66±2.90	9.86±3.61	16.2(NS)	12.5(NS)	10.2±3.9	18.1(NS)	8.45±1.69	5.86(NS)
μ_A	0.17±0.13^{A}	0.47±0.06^{B}	0.27±0.15^{A}	0.24±0.16^{A}	0.27±0.14^{A}	0.25±0.09^{A}	0.36±0.16^{A}	0.21±0.08^{A}	0.23±0.13^{A}	0.26±0.17^{A}	0.19(NS)	0.15(NS)	0.37±0.16^{A}	0.15(NS)	0.23±0.07^{A}	0.15±0.13^{A}
τ_A	25.7±9.8^{A}	24.4±6.3^{A}	19.3±3.0^{A}	17.9±3.2^{A}	18.0±2.8^{A}	17.1±3.0^{A}	14.8±2.0^{A}	17.5±3.9^{A}	16.2±4.2^{A}	17.6±6.5^{A}	26.8(NS)	25.6(NS)	15.6±5.1^{A}	31.2(NS)	17.3±4.9^{A}	18.9±10.8^{A}
t_{mA}	37.3±16.1^{A}	28.6±7.8^{A}	26.6±6.5^{A}	26.2±6.5^{A}	25.3±5.7^{A}	25.2±7.2^{A}	20.3±4.1^{A}	27.2±6.6^{A}	24.8±3.9^{A}	25.3±8.9^{A}	37.3(NS)	38.7(NS)	21.0±6.0^{A}	44.3(NS)	26.2±16.1^{A}	31.9±20.7^{A}
R^2	0.980	0.999	0.975	0.981	0.989	0.987	0.989	0.945	0.986	0.971	0.996	0.982	0.983	0.980	0.984	0.934

Table S7. Maximum and minimum values of productive yields of LAB bioproductions. In brackets, the media that generated the corresponding yields are also indicated.

Yields (units)	Lb 1	Lb2	Ln	Lb 3
$Y_{X/RS}$ (gX/gRS)	0.203 (Sk_Gr)<br>0.243 (Wh_Ha)	0.107 (He_Gu)<br>0.156 (Sk_Me)	0.079 (Sk_Ma)<br>0.176 (He_Po)	0.156 (Sk_Ma)<br>0.208 (Sk_Me)
$Y_{X/Pr}$ (gX/gPr)	1.84 (Sk_Me)<br>2.72 (Sk_Bo)	1.13 (He_Gu)<br>1.85 (MRS)	0.317 (Sk_RS)<br>1.89 (MRS)	1.56 (Sk_Ma)<br>2.46 (Sk_Gu)
$Y_{G/RS}$ (G/gRS)	0.513 (Sk_Gu)<br>0.680 (Wh_BW)	0.531 (He_RS)<br>0.716 (He_Ma)	0.510 (Sk_Bo)<br>2.10 (Sk_BW)	0.460 (He_BW)<br>0.568 (Sk_Po)
$Y_{G/Pr}$ (G/gPr)	4.88 (Sk_Gu)<br>8.19 (Sk_Bo)	5.39 (He_AHM)<br>7.97 (He_Po)	4.47 (Sk_RS)<br>11.10 (Sk_Gu)	4.42 (He_RS)<br>6.56 (MRS)
$Y_{L/RS}$ (gL/gRS)	0.586 (Sk_Bo)<br>0.719 (He_Gr)	0.685 (Wh_Gr)<br>0.899 (MRS)	0.702 (Sk_AHM)<br>1.53 (Sk_BW)	0.607 (Sk_BW)<br>0.748 (He_Gu)
$Y_{L/Pr}$ (gL/gPr)	5.69 (Wh_Ma)<br>8.21 (He_Gr)	6.65 (Wh_AHM)<br>9.96 (MRS)	3.01 (Sk_RS)<br>9.68 (Sk_Bo)	5.50 (Sk_BW)<br>9.21 (MRS)
$Y_{A/RS}$ (gA/gRS)	0.089 (He_RS)<br>0.142 (Sk_Po)	0.041 (MRS)<br>0.082 (Sk_Me)	0.017 (He_Bo)<br>0.094 (Sk_BW)	0.056 (He_AHM)<br>0.100 (Wh_AHM)
$Y_{A/Pr}$ (gA/gPr)	0.890 (He_RS)<br>1.43 (He_Ma)	0.453 (MRS)<br>0.884 (Sk_Me)	0.128 (Sk_RS)<br>0.572 (Sk_Me)	0.648 (He_AHM)<br>1.13 (Wh_Ma)

Table S8. Amino acids content (% or g/100 g total amino acids) of fish discards peptones (mean value ± confidence interval). OHPro: hydroxyproline.

	Sk_BW	Sk_RS	Sk_Ma	Sk_Po	Sk_Gu	Sk_Gr	Sk_Me	Sk_Ha	Sk_Bo	Sk_AHM	He_BW	He_RS	He_Ma	He_Po	He_Gu
**Asp**	9.77±0.05	9.64±0.05	9.13±0.03	9.61±0.08	9.12±0.50	9.46±0.10	7.61±0.78	8.84±0.52	8.86±0.02	7.98±0.22	9.85±0.10	10.13±0.04	9.84±0.32	9.95±0.08	9.88±0.20
**Thr**	4.45±0.19	4.18±0.06	4.36±0.08	3.47±0.11	4.02±0.11	3.75±0.04	4.13±0.42	4.02±0.12	4.12±0.03	4.00±0.02	4.16±0.14	4.27±0.06	4.02±0.39	4.37±0.23	4.29±0.06
**Ser**	5.81±0.10	5.50±0.09	5.38±0.11	5.15±0.01	5.86±0.11	6.08±0.32	5.39±0.44	5.82±0.05	4.69±0.08	5.85±0.07	5.38±0.25	5.12±0.07	5.12±0.36	5.17±0.49	5.26±0.66
**Glu**	14.45±0.24	13.50±0.01	12.88±0.26	12.55±0.18	12.49±0.01	13.06±0.31	11.84±0.69	12.45±0.04	12.41±0.11	12.36±0.33	15.01±0.61	13.83±0.08	15.32±0.57	15.40±2.48	16.33±0.67
**Gly**	10.88±0.30	12.36±0.35	11.64±0.07	11.46±0.16	15.05±0.08	14.76±0.06	16.12±0.10	16.23±0.44	11.37±0.10	15.94±0.32	7.18±0.34	8.29±0.16	6.99±0.48	7.44±1.83	6.73±0.45
**Ala**	8.25±0.33	8.47±0.19	8.21±0.05	8.10±0.05	8.85±0.10	8.94±0.13	8.65±0.63	9.26±0.08	7.85±0.06	9.01±0.15	6.35±0.01	7.68±0.05	6.23±0.31	6.64±0.67	6.50±0.40
**Cys**	0.35±0.03	0.31±0.01	0.26±0.01	0.33±0.00	0.26±0.02	0.31±0.02	0.33±0.01	0.25±0.01	0.44±0.03	0.29±0.06	0.39±0.09	0.36±0.01	0.41±0.06	0.39±0.01	0.36±0.04
**Val**	3.88±0.10	3.27±0.00	4.03±0.02	2.93±0.05	3.30±0.06	3.17±0.07	3.72±0.31	3.34±0.12	4.39±0.05	3.67±0.37	4.20±0.04	4.21±0.03	4.23±0.16	4.21±0.39	4.12±0.20
**Met**	3.22±0.35	3.24±0.13	3.05±0.11	3.31±0.12	3.30±0.26	3.09±0.06	2.00±0.17	2.60±0.01	3.11±0.04	2.12±0.08	3.56±0.08	3.66±0.02	3.69±0.18	3.27±0.12	3.47±0.50
**Ile**	2.96±0.12	2.42±0.05	3.13±0.02	2.06±0.07	2.06±0.17	1.95±0.01	2.05±0.04	1.80±0.03	3.44±0.05	2.00±0.13	3.37±0.74	3.33±0.03	3.40±0.58	3.94±1.06	3.80±1.34
**Leu**	6.76±0.05	6.13±0.09	6.83±0.07	5.87±0.06	5.72±0.10	6.03±0.01	5.62±0.07	5.55±0.12	6.57±0.01	5.68±0.09	7.10±0.11	7.41±0.03	7.14±0.07	7.59±0.28	7.52±0.04
**Tyr**	2.87±0.03	2.76±0.01	2.81±0.05	2.64±0.08	2.29±0.03	2.15±0.10	2.91±0.05	2.42±0.09	2.69±0.02	2.81±0.12	3.72±0.29	3.13±0.00	4.13±0.39	3.42±0.11	3.77±0.58
**Phe**	3.97±0.17	3.98±0.03	3.86±0.19	4.00±0.03	3.70±0.25	4.29±0.21	4.63±0.70	3.67±0.09	4.12±0.02	4.05±0.40	4.16±0.18	4.79±0.04	4.49±0.81	3.86±0.69	3.26±0.50
**His**	1.95±0.00	1.80±0.00	2.72±0.25	1.93±0.02	1.91±0.05	2.03±0.36	2.44±0.51	2.18±0.09	1.35±0.12	2.29±0.24	2.22±0.41	1.95±0.01	2.14±0.69	2.07±0.56	2.23±0.18
**Lys**	6.77±0.13	6.73±0.06	6.56±0.07	6.21±0.33	5.93±0.32	5.80±0.25	6.11±0.77	5.37±0.70	6.33±0.04	5.94±0.48	7.82±0.06	7.09±0.06	7.59±0.10	7.04±0.45	7.03±0.43
**Arg**	6.25±0.05	6.63±0.09	6.52±0.11	5.85±0.18	6.66±0.42	6.42±0.09	6.74±0.76	6.34±0.06	6.08±0.02	6.47±0.27	7.01±0.15	6.10±0.03	6.78±1.19	6.23±0.10	6.31±0.26
**OHPro**	1.99±0.31	2.78±0.16	2.21±0.27	9.41±1.28	3.24±0.38	2.55±0.01	7.69±1.13	3.79±0.19	6.40±0.04	3.60±0.42	4.14±0.42	4.42±0.39	3.98±0.22	4.08±0.42	4.28±0.04
**Pro**	5.43±0.01	6.31±0.08	6.45±0.21	5.11±0.29	6.24±0.48	6.18±0.04	0.36±0.08	6.06±0.11	5.80±0.07	5.93±0.14	4.37±0.61	4.22±0.02	4.46±0.81	4.92±0.02	4.89±0.03

Table S9. Continuation of Table S8.

	He_Gr	He_Me	He_Ha	He_Bo	He_AHM	Wh_BW	Wh_RS	Wh_Ma	Wh_Po	Wh_Gu	Wh_Gr	Wh_Bo	Wh_Ha	Wh_Me	Wh_AHM
**Asp**	9.89±0.06	9.54±0.57	9.90±0.01	10.04±0.15	9.45±0.32	10.39±0.10	10.23±0.07	9.86±0.10	10.02±0.02	9.38±0.52	10.52±0.04	9.69±0.22	11.48±0.27	11.21±0.21	10.01±0.36
**Thr**	4.08±0.04	4.21±0.28	4.42±0.15	4.30±0.04	4.29±0.08	4.40±0.16	4.99±0.07	4.73±0.13	4.45±0.17	4.62±0.20	4.73±0.15	4.74±0.11	4.24±0.33	4.05±0.27	4.49±0.82
**Ser**	5.32±0.23	5.28±0.60	5.36±0.10	5.16±0.61	5.34±0.15	5.13±0.13	5.02±0.02	4.82±0.18	4.77±0.06	4.31±0.19	4.58±0.15	4.95±0.08	5.15±0.08	5.45±0.01	4.96±0.24
**Glu**	13.82±0.05	16.49±0.79	14.03±0.25	15.36±0.46	14.12±0.38	14.90±0.27	15.81±0.37	13.39±0.49	13.87±0.10	13.74±0.52	14.77±0.24	13.82±0.41	16.80±0.06	15.63±0.10	13.84±0.51
**Gly**	8.52±0.06	6.90±0.73	8.27±0.11	7.11±0.02	6.73±0.23	5.96±0.18	5.92±0.05	5.78±0.15	6.17±0.02	8.63±1.92	4.81±0.03	7.22±0.03	4.75±0.07	7.02±0.07	6.27±0.11
**Ala**	7.00±0.13	6.51±0.75	7.03±0.21	6.37±0.14	6.57±0.05	7.26±0.14	7.25±0.06	6.98±0.05	7.17±0.12	6.96±0.34	6.79±0.11	6.52±0.15	6.81±0.22	7.45±0.19	6.92±0.59
**Cys**	0.45±0.02	0.33±0.12	0.40±0.03	0.39±0.01	0.45±0.00	0.54±0.02	0.41±0.02	0.61±0.04	0.55±0.03	0.85±0.17	0.76±0.07	0.70±0.06	0.56±0.10	0.50±0.03	0.53±0.04
**Val**	4.28±0.02	4.17±0.09	4.38±0.05	4.23±0.03	4.39±0.10	4.52±0.02	4.00±0.08	5.25±0.07	4.50±0.11	4.16±0.10	4.75±0.07	4.76±0.16	3.65±0.11	3.51±0.06	4.22±0.87
**Met**	3.65±0.05	3.45±0.53	3.41±0.23	3.78±0.25	3.93±0.64	3.63±0.15	2.61±0.07	3.57±0.17	3.54±0.19	3.06±0.25	3.71±0.18	3.31±0.09	3.89±0.15	3.77±0.22	3.21±0.56
**Ile**	3.32±0.04	3.91±1.40	3.41±0.08	3.22±0.20	3.53±0.35	3.72±0.07	3.71±0.11	4.32±0.04	3.75±0.14	4.01±0.16	4.10±0.04	4.09±0.16	2.94±0.31	2.68±0.18	3.45±0.98
**Leu**	7.43±0.03	7.80±0.81	7.45±0.08	6.89±0.25	7.12±0.34	8.36±0.07	7.89±0.06	8.34±0.07	7.97±0.03	7.60±0.38	8.57±0.04	7.56±0.01	8.01±0.19	7.53±0.09	7.65±0.27
**Tyr**	3.28±0.08	3.72±0.62	3.33±0.05	4.29±1.35	4.78±0.78	3.57±0.16	2.94±0.03	3.47±0.06	3.56±0.07	3.42±0.41	3.69±0.06	3.61±0.10	3.84±0.10	3.53±0.10	3.38±0.05
**Phe**	4.55±0.06	3.28±0.30	4.40±0.20	4.10±0.34	4.38±0.31	4.79±0.19	4.12±0.06	4.58±0.23	4.66±0.15	4.55±0.23	4.94±0.19	4.56±0.23	4.69±0.27	4.76±0.21	4.49±0.37
**His**	1.55±0.08	2.21±0.29	1.72±0.12	2.39±0.26	2.57±0.07	2.02±0.07	2.62±0.02	4.34±0.13	3.23±0.10	2.39±0.29	2.37±0.08	2.37±0.02	2.46±0.23	2.51±0.17	2.99±0.02
**Lys**	7.08±0.07	6.92±0.25	7.25±0.18	7.53±0.18	7.48±0.51	8.52±0.11	8.86±0.05	7.72±0.13	8.39±0.08	7.19±0.62	8.28±0.20	7.68±0.07	8.91±0.45	7.70±0.29	8.68±0.16
**Arg**	6.55±0.06	6.24±0.13	6.31±0.08	6.49±0.34	6.76±0.31	6.00±0.08	6.37±0.06	5.41±0.10	6.14±0.08	6.71±0.32	5.46±0.07	6.21±0.06	5.58±0.34	5.76±0.21	6.29±0.23
**OHPro**	4.82±0.25	4.17±0.17	4.15±0.73	3.49±0.47	3.50±0.42	3.04±0.52	2.94±0.29	3.14±0.25	3.31±0.28	2.97±0.21	3.74±0.07	3.41±0.11	2.81±0.48	2.96±0.24	3.68±1.23
**Pro**	4.42±0.26	4.87±0.13	4.77±0.33	4.85±0.52	4.62±0.13	3.72±0.28	4.48±0.04	3.68±0.22	4.07±0.26	5.63±0.77	3.48±0.10	4.74±0.05	3.45±2.21	4.04±0.23	4.19±0.42
